# GIPC1 Restrains the Progression and Chemoresistance of Colorectal Cancer by Regulating TTC7B/mTOR/NF-κB Axis

**DOI:** 10.7150/ijbs.119064

**Published:** 2026-01-01

**Authors:** Dongxue Gan, Cheng Yang, Xiangjing Shen, Jingjie Shi, Ronglin Wang, Liaoliao Zhu, Hong Li, Jing Luo, Ting Zhao, Junqiang Li, Yang Song, Haichuan Su

**Affiliations:** Department of Oncology, Tangdu Hospital, Fourth Military Medical University, State Key Laboratory of Holistic Integrative Management of Gastrointestinal Cancers, Xi'an, Shaanxi 710038, China.

**Keywords:** colorectal cancer, GIPC1, ubiquitination, TTC7B, chemoresistance

## Abstract

Colorectal cancer (CRC) remains a leading cause of cancer-related mortality, highlighting the need for a deeper understanding of its molecular mechanisms to drive the development of novel therapeutic approaches. In this study, the findings indicated a significant reduction in PDZ Domain Containing Family Member 1 (GIPC1) expression in CRC tissues, which correlated with poor prognosis in patients with CRC at pathological stages T1 and T2. GIPC1 acted as a tumor suppressor gene that inhibited CRC cell proliferation, colony formation, migration, and invasion. Additionally, it enhanced CRC cell sensitivity to first-line chemotherapies such as 5-fluorouracil (5-FU), oxaliplatin (OXA), and irinotecan (CPT-11). Mechanistically, GIPC1 reduced the ubiquitination level of tetratricopeptide repeat domain 7B (TTC7B) by downregulating the E3 ubiquitin ligase TRIM21, thereby stabilizing TTC7B's expression and inhibiting the downstream mTOR/NF-κB signaling cascade. Moreover, *in vivo* studies confirmed the inhibitory role of GIPC1 in CRC growth and found that GIPC1-loaded lipid nanoparticles (GIPC1-LNPs) combined with 5-FU treatment had a more significant antitumor effect. In conclusion, this study reveals the GIPC1/TRIM21/TTC7B/mTOR/NF-κB tumor-suppressive axis in CRC and highlights the potential of GIPC1 for early diagnosis and overcoming chemoresistance in CRC patients.

## Introduction

Colorectal cancer (CRC) represents a considerable challenge to global health and remains a primary contributor to cancer-associated deaths 1. Current clinical treatments for CRC primarily include surgical resection, chemotherapy, and targeted therapies. Despite advances in early diagnosis and therapeutic strategies, the prognosis for advanced CRC remains poor 2,3. Consequently, further exploration of the molecular mechanisms driving CRC progression is essential.

Chemoresistance is a significant obstacle in CRC treatment. Studies have shown that tumor-associated macrophages (TAMs) contribute to 5-FU resistance under hypoxic conditions by overexpressing dihydropyrimidine dehydrogenase (DPD) 4. Additionally, upregulation of ubiquitin-conjugating enzyme E2T (UBE2T) has been associated with chemoresistance in CRC, as UBE2T enhances Wnt/β-catenin signaling, promoting resistance 5. Moreover, increased LGR4 expression and activation of the Wnt pathway have been identified as key mechanisms underlying chemoresistance 6. These findings offer valuable insights into the molecular basis of chemoresistance in CRC.

GIPC1, a widely expressed PDZ protein, interacts with numerous binding partners, predominantly transmembrane receptors, adhesion molecules, and proteins involved in endocytosis and intracellular transport 7-9. The N-terminus (GH1) and C-terminus (GH2) of GIPC1 each contain GIPC homologous domains. The GH1 domain facilitates self-dimerization, while the GH2 domain is associated with retrograde actin movement and the internalization of endocytic vesicles, a process driven by Myosin 6 (MYO6) 10. Numerous studies have highlighted the pivotal role of GIPC1 in various malignancies. In breast cancer, GIPC1 modulates cell morphology and migration through its interaction with MYO6 11. In non-small cell lung cancer, GIPC1 regulates the endocytosis of nerve growth factor and integrins *via* its interaction with SH3BP4, influencing cellular responses to the microenvironment 12. Additionally, GIPC1 expression correlates with metastasis formation and non-metastatic survival in patients with gastric cancer 13. In MACC1-driven CRC, GIPC1 acts as protein interaction partner and as transcription factor of MACC1, playing a dual role in tumor progression and metastasis 14. In cases of cervical cancer linked to HPV-18 infection, GIPC1 is significantly downregulated. This downregulation leads to resistance to the inhibitory signaling pathways typically mediated by TGF-β, which resistance results from the instability of the TGF-βR3 15. In pancreatic cancer cells, the absence of GIPC promotes the depletion of the drug resistance molecule ABCG2 through exosome-mediated exocytosis or causes the sequestering of ABCG2 in vesicles, rendering it nonfunctional, which then makes cancer cells sensitive to gemcitabine 16. However, the specific role of GIPC1 in CRC chemoresistance remains poorly understood.

TTC7B, a member of the tetratricopeptide repeat (TPR) gene family, is involved in the biosynthesis of phosphatidylinositol phosphates 17. Research on TTC7B's function in tumor progression is limited. Bioinformatics analyses have suggested that TTC7B may serve as a novel prognostic biomarker in head and neck squamous cell carcinoma and cervical cancer 18,19. Ren et al. demonstrated that in colon cancer, TTC7B triggers the RXRA-FTO axis by binding to PI4KA, reducing m6A modification of total RNA and inhibiting colon cancer cell proliferation 20.

This study examined the role of GIPC1 in CRC progression and chemoresistance. GIPC1 inhibits the mTOR/NF-κB signaling pathway by stabilizing TTC7B expression, thereby restraining CRC chemoresistance and progression. Notably, GIPC1-loaded lipid nanoparticles (GIPC1-LNPs) exhibited significant antitumor effects in a CRC resistance model. These findings highlight the critical role of GIPC1 in CRC chemoresistance and tumor progression, suggesting a promising combination therapy to overcome chemoresistance in CRC.

## Materials and Methods

### Cell line culture

Human CRC cell lines (DLD1, SW480, HCT116) were sourced from Procell Life Science & Technology Company (Wuhan, China) and authenticated by STR profiling. DLD1 cells were cultured in complete RPMI 1640 medium (HyClone, Utah, USA), SW480 cells in complete L-15 medium (Gibco BRL, Rockville, MD), and HCT116 cells in complete McCoy's 5A medium (HyClone, Utah, USA). All media contained 10% fetal bovine serum (FBS) (HyClone, Utah, USA), penicillin (100 U/mL), and streptomycin (100 U/mL). Cells were maintained in a 37 °C, 5% CO2 incubator under standard cultivation conditions.

### Animal studies

Male BALB/c nude mice, 4-6 weeks old (18-20 g), were obtained from the Experimental Animal Center of the Fourth Military Medical University and housed in a specific pathogen-free (SPF) environment. To establish a xenograft tumor model, 4 × 10^6^ DLD1 cells were subcutaneously injected into the mice. Tumor volume was measured every other day, starting seven days post-injection. For 5-FU treatment, mice were administered 25 mg/kg of 5-FU *via* intraperitoneal injection every other day. For lipid nanoparticles (LNPs) treatment, 25 mg/kg of LNPs were injected *via* the vein every other day. The control group received only carrier injections. Mice were euthanized, and tumors and organs (heart, liver, spleen, lung, and kidney) were harvested for analysis. Tumor volume was calculated using the formula: Tumor volume = 0.5 × longest diameter × shortest diameter^2^. All animal procedures were approved by the Animal Ethics Committee of Tangdu Hospital, Fourth Military Medical University.

### Patient's specimens

Tissues obtained from CRC patients, alongside adjacent non-malignant tissues, were collected from individuals undergoing surgery at Tangdu Hospital, affiliated with the Fourth Military Medical University (Xi'an, China). Ethical approval for all protocols was granted by the Ethics Committee of the Fourth Military Medical University, and written informed consent was obtained from all participants. The CRC tissue microarray (NO. HColA180Su21) was purchased from Shanghai Outdo Biotechnology Co., Ltd, and relevant clinical and pathological data were collected with written informed consent. Data from TCGA and GEO databases are provided in Supporting Information Table S1.

### Lentivirus infection

Following the manufacturer's guidelines, expression vector and packaging plasmids (pMD2G and psPAX2) were co-transfected into 293T cells utilizing the HighGene transfection reagent (RM09014, Abclonal, Wuhan, China) to generate lentivirus. Lentivirus was then transfected into CRC cells with polybrene (40804ES76, Yasen, Shanghai, China). After 24 hours, stable transfected cell lines were selected using puromycin (13884, Cayman, USA) or G418 (Sigma-Aldrich, St. Louis, MO, USA), and the expression of target genes was assessed at designated time points by Western blotting. The shRNA sequences and overexpression RNA used in this study are detailed in Supporting Information Tables S2 and S3.

### Cell viability assay

Cell viability was assessed following established protocols 21. Briefly, DLD1, SW480, and HCT116 cells were seeded at 1 × 10^3^ cells per well in 96-well plates. After 24 hours, cells were treated with 5-FU (5, 10, or 20 μg/mL), OXA (25, 50, or 100 μM), or CPT-11 (10 or 20 μM) for 48 hours. Cell viability was measured using the CCK8 assay, with absorbance readings taken at 450 nm. Cell viability was determined as follows: Cell viability (%) = (A450 of treated cells) / (A450 of untreated cells).

### Cell proliferation assay

The CCK8 and colony formation assays were performed as described previously 22. The CCK8 reagent (KeyGEN, Jiangsu, China) was used in accordance with the manufacturer's instructions. DLD1, SW480, and HCT116 cells were plated at 1 × 10^3^ cells per well in 96-well plates, and absorbance was recorded at 450 nm at 0, 24, 48, 72, and 96-hour intervals to assess cell proliferation. For the colony formation assay, cells were seeded at a density of 500 cells per well in 6-well plates, with medium changes every 3 days, and cultured for 2 weeks. After incubation, colonies were fixed in 4% paraformaldehyde, stained with a 0.1% crystal violet solution, photographed, and counted.

### Transwell assay

Cell migration and invasion assays were conducted as previously described 23. DLD1, SW480, and HCT116 cells were starved overnight, then seeded at 2 × 10^4^ cells per well in transwell chambers, pre-treated with or without 50 µL of Matrigel (1 mg/mL) for migration or invasion assays. The lower chamber was contained complete medium supplemented 10% FBS. After 48 hours of incubation, cells that migrated to the bottom surface were fixed with 4% paraformaldehyde, stained with 0.1% crystal violet solution, and visualized. Non-migrating cells on the upper surface of the membrane were meticulously removed with a cotton swab. Migrating cells were observed and photographed under an inverted microscope (Olympus IX-71, Tokyo, Japan), followed by counting.

### Western blotting

Western blotting was performed according to established protocols 23. Bands of interest were visualized using enhanced chemiluminescence on a BIO-RAD ChemiDoc XRS+ imaging system. Antibodies used in this study are listed in Supporting Information Table S4.

### GST pull-down assay

GST-tagged proteins were purified using the GST tag protein purification kit (P2262, Biyuntian, Wuhan, China) following the manufacturer's instructions. GIPC1-GST or GST alone was purified from E. coli strain BL21. TTC7B-GFP, extracted from 293T cells, was incubated with either GST or GIPC1-GST beads at 4°C for 5 hours. Following incubation, the beads underwent three washing cycles with PBS, and immunoblotting (IB) analysis was performed to assess the interactions.

### Immunofluorescence (IF)

293T cells (1 × 10^4^) were seeded in laser confocal dishes. After 4 hours of transfection with GIPC1-Flag and TTC7B-GFP plasmids, cells were incubated for an additional 48 hours. Cells were washed three times with PBS, fixed with 4% formaldehyde for 10 minutes, and permeabilized with 0.5% Triton X-100 for 5 minutes. Blocking was performed with 5% BSA for 30 minutes. Primary antibody anti-GIPC1 rabbit (14822-1-AP, Proteintech, China), was applied, followed by Alexa Fluor 647-labeled anti-rabbit secondary antibody (ab150083, Abcam, USA) and DAPI (C1005, Beyotime, China) for nuclear staining. Image acquisition was performed using a confocal microscope (STELLARIS 5, Leica, Germany).

### Hematoxylin and Eosin (H&E)

Various murine organs, including the heart, liver, spleen, lung, and kidney, and tumor tissues were collected and fixed overnight in 4% paraformaldehyde after the mouse studies. The tissues were then embedded in paraffin wax. Histological sections (4 µm in thickness) were prepared and stained with H&E. Images were captured and recorded using a microscope (DM4000b, Leica, Germany).

### Immunohistochemistry (IHC)

For IHC staining, a CRC patient-specific tissue microarray kit (HColA180Su21, Outdo Biotech, Shanghai, China) was used. The staining protocol followed previously established methods 22. Antibodies for GIPC1, TTC7B, and Ki67 (GB111499, Servicebio, China) were applied.

### Transmission Electron Microscopy (TEM)

In TEM, synthesized liposome nanoparticles (10 mL) were placed on a copper grid and incubated for 3-5 minutes. Excess liquid was subsequently removed using filter paper. Next, 10 µL of phosphotungstic acid was added and left for 5 minutes before air drying. Samples were observed and recorded under a transmission electron microscope (TF20, FEI, USA).

### Formation and evaluation of GIPC1-LNPs

GIPC1-LNPs were encapsulated in a lipid membrane using incubation and extrusion techniques. The mixture was co-extruded 20 times through a 200 nm polycarbonate membrane to generate GIPC1-LNPs. Particle size, polydispersity index (PDI), and zeta potential were evaluated employing a nanoparticle potentiometer (NanoBrook 90plus PALS, Brookhaven, USA).

### Statistical analysis

Each experiment included a control group and experimental groups, with all experiments conducted independently at least three times. Data are presented as mean ± SD. Statistical analyses were conducted using SPSS 19.0 (SPSS Inc, Chicago, Illinois, USA). Student's t-test or one-way ANOVA was used to determine statistical differences between groups. Kaplan-Meier curves assessed the association between mRNA/protein levels and overall survival (OS). Receiver operating characteristic (ROC) curves were used to evaluate the diagnostic significance of GIPC1. Statistical significance was defined as *p < 0.05, **p < 0.01, and ***p < 0.001. ns, not significant.

## Results

### GIPC1 expression is reduced and correlates with unfavorable outcomes in CRC patients

**Figure 1:**
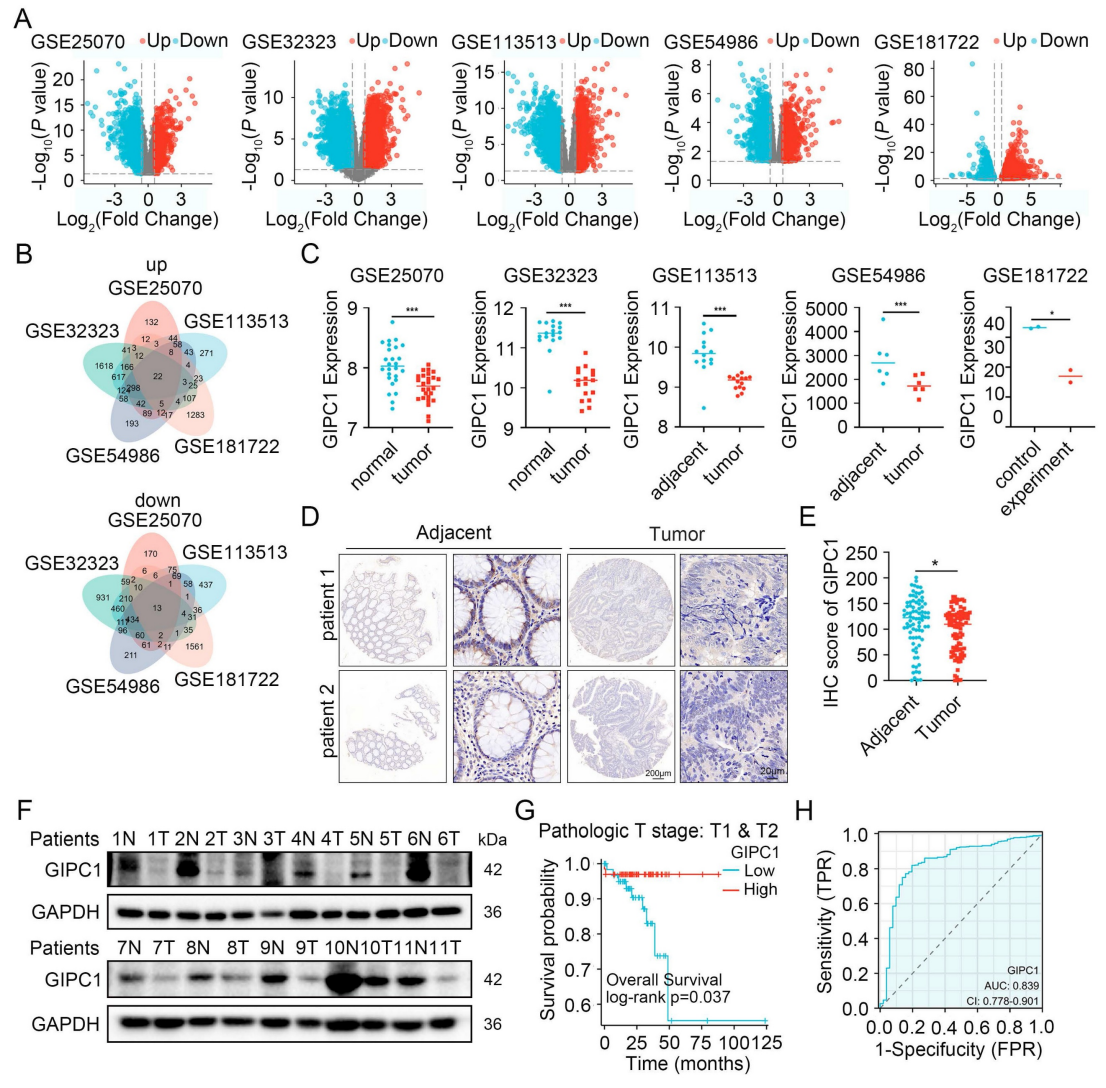
GIPC1 expression is diminished in colorectal cancer and correlates with unfavorable prognosis. (A) Volcano plots illustrate differentially expressed genes (DEGs) from the datasets GSE25070, GSE32323, GSE11353, GSE54986, and GSE181722. (B) Venn diagrams illustrating the overlapping DEGs across the datasets GSE25070, GSE32323, GSE11353, GSE54986, and GSE181722. (C) mRNA expression levels of GIPC1. (D) IHC staining detecting GIPC1 expression in colorectal cancer tissue microarray (TMA), which includes matched adjacent non-malignant tissue and colorectal cancer tissue. Scale bars are shown in Figure 1. (E) Scoring of GIPC1 expression in IHC data (n = 94). (F) Protein expression levels of GIPC1 in CRC tissue compared to matched normal tissue adjacent cancer (n = 11). (G) Kaplan-Meier survival analysis examining the relationship between GIPC1 expression and OS in CRC patients with pathological stages T1 and T2 in the TCGA database. (H) Receiver operating characteristic (ROC) curve evaluating the diagnostic value of GIPC1 for CRC. Data are presented as mean ± SD. *P < 0.05, **P < 0.01, ***P < 0.001.

CRC remains one of the predominant causes contributing to cancer-associated mortality 1. To uncover the molecular mechanisms driving CRC progression, several datasets (GSE25070, GSE32323, GSE113513, GSE54986, and GSE181722) were analyzed (Figure 1A), identifying 22 genes with significant upregulation and 13 with significant downregulation (Figure 1B). Among these genes, GIPC1 has attracted our attention. GIPC1 expression was notably downregulated in CRC tissues (Figure 1C). To further investigate GIPC1's role in CRC, pan-cancer analysis using The Cancer Genome Atlas (TCGA) database revealed a significant reduction of GIPC1 expression in colon cancer (COAD) and rectal adenocarcinoma (READ) (Figure S1A-B). This was confirmed at the protein level by immunohistochemistry (IHC). IHC analysis of tissue microarrays from CRC patients and collected colorectal cancer (CRC) samples and adjacent normal tissues, showed decreased GIPC1 expression in CRC tissues compared to non-malignant tissues (Figure 1D-E and Figure S1C). Western blotting also confirmed the reduced GIPC1 expression in tumor tissues, with higher levels in normal tissues adjacent cancer (Figure 1F and Figure S1D). Additionally, data from the HPA and CPTAC datasets supported these findings (Figure S1E-F). To assess the clinical significance of GIPC1, Kaplan-Meier survival analysis showed a significant association between reduced GIPC1 expression and poor OS in CRC patients with pathological stages T1 and T2 in the TCGA database (Figure 1G), suggesting that GIPC1 may function as a tumor suppressor gene (TSG) in early CRC. Furthermore, ROC curve analysis demonstrated that GIPC1 expression has diagnostic value for CRC in the TCGA dataset, with an AUC of 0.839 (Figure 1H). These findings highlight the critical involvement of GIPC1 in CRC.

### GIPC1 inhibits chemoresistance, growth, and metastasis in CRC

**Figure 2:**
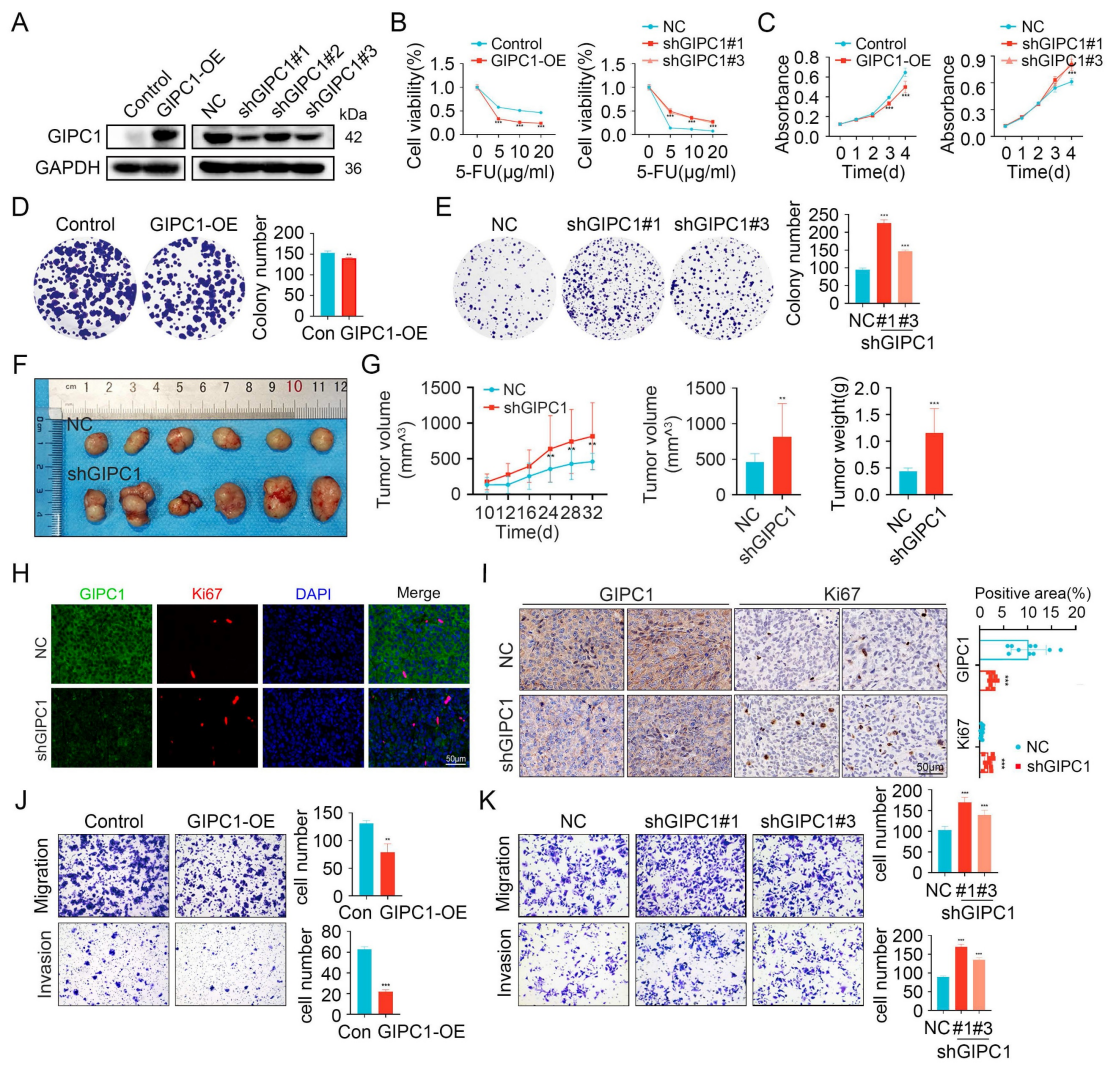
GIPC1 inhibits chemotherapy resistance, growth, and metastasis. (A) Overexpression of GIPC1 in DLD1 cells (left) and knockdown of GIPC1 using three independent shRNAs in DLD1 cells (right). (B) Viability of DLD1 cells after exposure to varying concentrations of 5-FU. (C-E) CCK8 and colony formation assays demonstrating the proliferation ability of DLD1 cells. (F) Representative images of tumors from each mouse group (n = 6). DLD1 cells were injected subcutaneously into nude mice. (G) Tumor volume and weight were measured (n = 6). (H-I) Immunofluorescence (IF, H) and immunohistochemistry (IHC, I) staining of tumor sections from various groups. Scale bar = 50 µm. (J-K) Transwell assays assessing the migration and invasion capabilities. Data are presented as mean ± SD. **P < 0.01, ***P < 0.001.

To explore the role of GIPC1 in chemoresistance and tumor progression, we measured its expression levels in colorectal cancer cell lines. The results showed that GIPC1 is generally expressed at low levels in CRC cell lines (Figure S1G). Then we engineered GIPC1 overexpression in colorectal cancer cell lines (DLD1, SW480, and HCT116), followed by GIPC1 knockdown in these cell lines (Figure 2A and Figure S2A-B). Chemoresistance is a major contributor to poor prognosis in CRC, and the impact of GIPC1 manipulation on drug sensitivity was evaluated. Overexpression of GIPC1 increased the sensitivity of DLD1, SW480, and HCT116 cells to common chemotherapeutic agents, including 5-fluorouracil (5-FU), oxaliplatin (OXA), and irinotecan (CPT-11). In contrast, GIPC1 knockdown reduced sensitivity to these drugs in the same cell lines (Figure 2B, Figure S2C-D and Figure S3). CCK8 and colony formation assays revealed that GIPC1 overexpression suppressed cellular proliferation, whereas GIPC1 knockdown enhanced cell proliferation (Figure 2C-E and Figure S2E-H). Additionally, a subcutaneous tumor model in nude mice showed accelerated tumor growth upon GIPC1 knockdown (Figure 2F-I). Transwell assays further indicated that GIPC1 overexpression inhibited cell migration and invasion, while GIPC1 knockdown promoted migration and invasion (Figure 2J-K and Figure S2I-L). Collectively, these findings suggest that GIPC1 functions as a TSG in CRC.

### GIPC1 interacts with TTC7B

**Figure 3:**
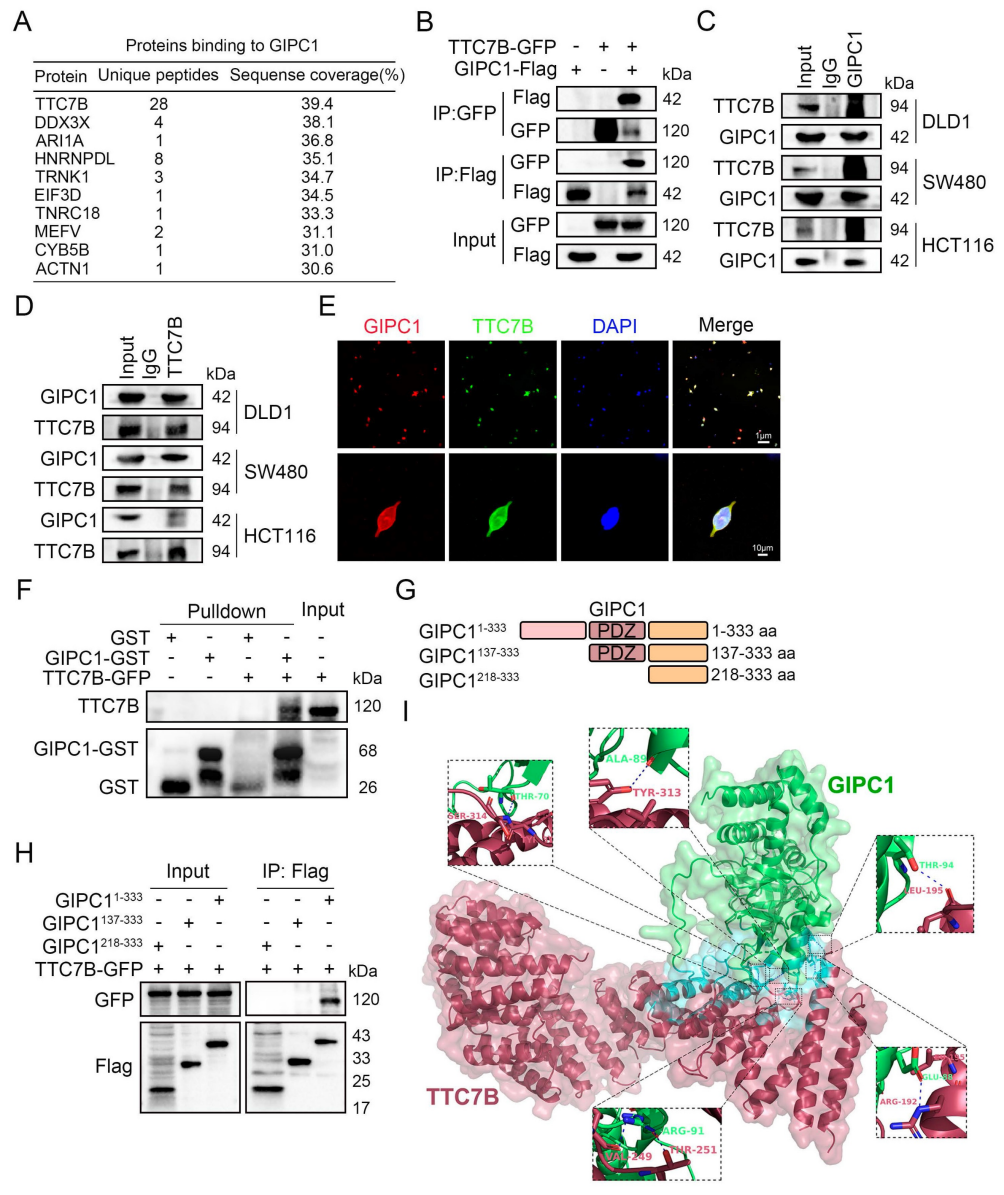
GIPC1 interacts with TTC7B. (A) GIPC1 interacting proteins. (B) Overexpression of GIPC1-Flag and/or TTC7B-GFP in 293T cells, followed by Co-IP of cell lysates using anti-DDDDK-Tag (Flag) or anti-GFP-Tag antibodies. (C-D) Co-IP confirming the interaction between GIPC1 and TTC7B. (E) IF assessment of GIPC1 and TTC7B localization in cells. Scale bars are shown in Figure 3. (F) Exogenous GIPC1 interacts with TTC7B. Purified TTC7B-GFP from 293T cells was incubated with either purified recombinant GIPC1-GST or GST. After GST pull-down assays, the interaction between TTC7B protein and GIPC1 was analyzed. (G) GIPC1 fragments were used in experiments. (H) GIPC1-Flag fragments and TTC7B-GFP were co-transfected into 293T cells, followed by Co-IP using Flag beads to isolate the proteins. (I) Diagram illustrating the predicted binding sites between GIPC1 and TTC7B.

Elucidate the functional mechanism of GIPC1 in CRC cells, co-immunoprecipitation (Co-IP) combined with mass spectrometry was utilized to identify proteins interacting with GIPC1. We prepared cell lysates from wild-type DLD1 cells were and subjected them to immunoprecipitation. Then, followed by SDS-PAGE was performed to separate the immunoprecipitated proteins. We stained the gel with Coomassie blue to visualize protein bands, which were then excised and analyzed by matrix-assisted laser desorption/ionization time-of-flight mass spectrometry to identify GIPC1 binding proteins. Mass spectrometry results identified TTC7B in the GIPC1 immunoprecipitates, exhibiting substantial sequence coverage (Figure 3A and Table S5). To validate this interaction, Co-IP experiments were conducted, in which exogenous GIPC1-Flag and TTC7B-GFP were overexpressed in 293T cells. TTC7B was immunoprecipitated using Flag antibodies, and GIPC1 was immunoprecipitated with GFP antibodies. The interaction between GIPC1 and TTC7B was confirmed (Figure 3B). This interaction was further validated in DLD1, SW480, and HCT116 cells (Figure 3C-D). To examine the subcellular localization of GIPC1 and TTC7B, confocal microscopy with immunofluorescence (IF) staining was performed on 293T cells, revealing co-localization of GIPC1 and TTC7B (Figure 3E), providing a spatial basis for their interaction. Additionally, GST-pulldown assay confirmed a direct interaction between GIPC1 and TTC7B (Figure 3F).

To identify which structural domain of GIPC1 interacts with TTC7B, we assessed the ability of various GIPC1 fragments to precipitate TTC7B (Figure 3G). The results showed that the GIPC1 region from amino acids 1 to 137, excluding the PDZ domain, binds TTC7B (Figure 3H). Subsequently, to pinpoint the precise binding sites on GIPC1 involved in this interaction, we used the HDOCK Server to predict protein-protein binding sites, then visualized the results. The prediction revealed 5 distinct binding sites (Figure 3I).

### GIPC1 improves TTC7B protein stability by downregulating the expression of E3 ubiquitin ligase TRIM21 to reduce ubiquitination of TTC7B protein

**Figure 4:**
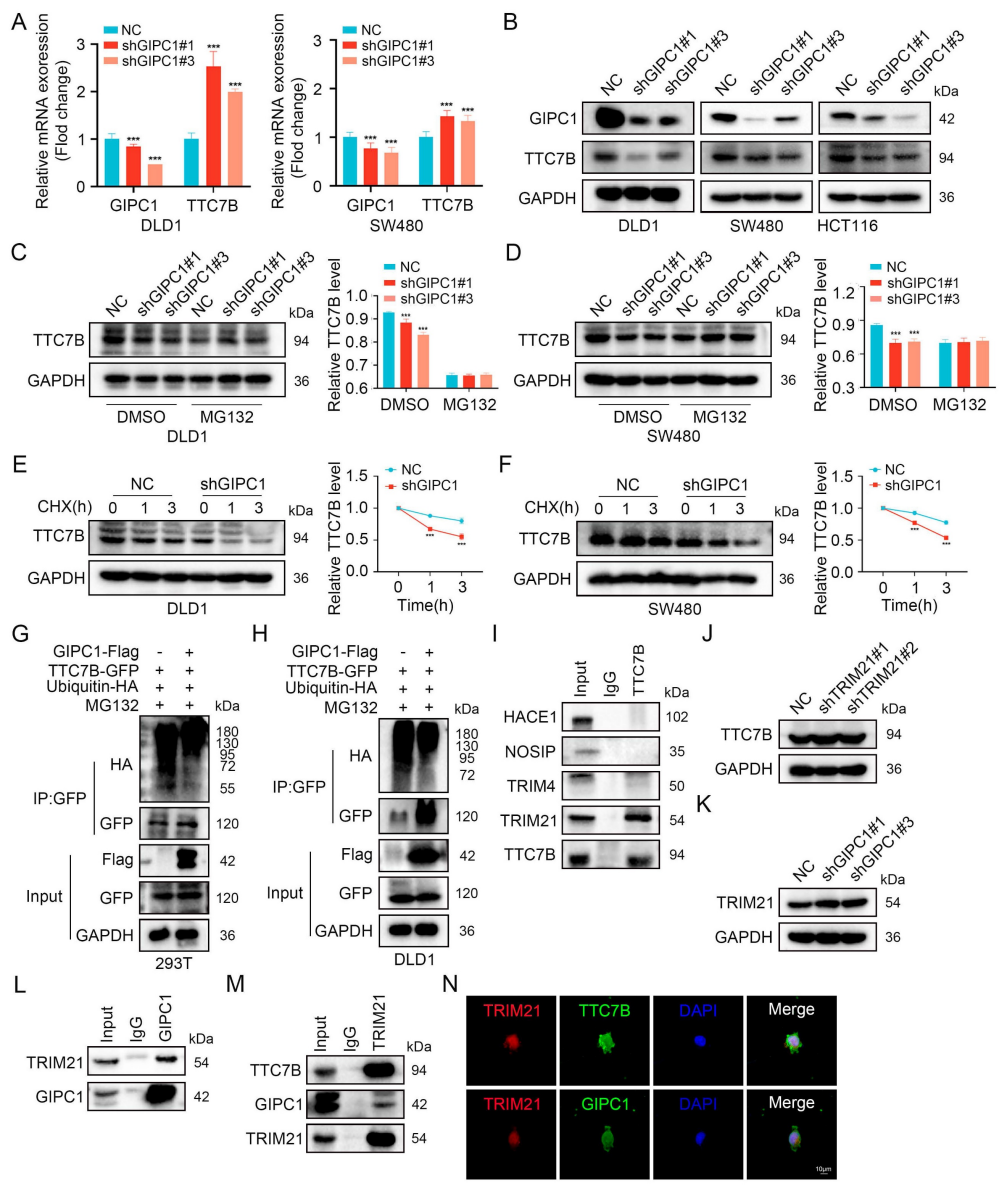
GIPC1 stabilizes TTC7B protein by downregulating TRIM21 expression to reduce TTC7B ubiquitination. (A) qRT-PCR analysis of GIPC1 mRNA and TTC7B mRNA expression in GIPC1 knockdown DLD1 and SW480 cells. (B) Knockdown of GIPC1 in colorectal cancer cells, followed by examination of TTC7B protein levels. (C-D) DLD1 and SW480 cells treated with or without MG132 (20 µM) for 4 hours, followed by analysis of TTC7B expression. (E-F) DLD1 and SW480 cells treated with cycloheximide (CHX, 100 mg/mL) for varying time intervals, followed by assessment of TTC7B protein levels. Quantitative analysis of TTC7B expression relative to GAPDH. (G-H) GIPC1 inhibits TTC7B ubiquitination. Co-transfection of TTC7B-GFP, ubiquitin-HA, and/or GIPC1-Flag into 293T and DLD1 cells, treated with MG132 (20 μM) for 4 hours. Analysis of TTC7B ubiquitination levels. (I) Co-IP validating the interaction between TTC7B and Screened E3 ubiquitin ligases. (J) Analysis of TTC7B expression in TRIM21 knockdown DLD1 cells. (K) Analysis of TRIM21 expression in GIPC1 knockdown DLD1 cells. (L) Co-IP validating the interaction between GIPC1 and TRIM21. (M) Verify the interaction of TRIM21 with GIPC1 and TTC7B, respectively. (N) Cellular IF evaluating the localization of TRIM21 with GIPC1 and TTC7B in cells, respectively. Scale bar = 10 µm. Data are presented as mean ± SD. ***P < 0.001.

We examined whether GIPC1 affects TTC7B expression to determine if TTC7B is a substrate of GIPC1. TTC7B mRNA levels were analyzed, revealing that GIPC1 negatively regulated TTC7B expression at the transcriptional level (Figure 4A). In CRC cells, GIPC1 knockdown led to decreased TTC7B protein levels (Figure 4B). To investigate the mechanism behind TTC7B degradation, cells were treated with cycloheximide (CHX), a protein synthesis inhibitor, together with the proteasome inhibitor MG132. In GIPC1 knockdown cells, MG132 treatment restored TTC7B protein levels (Figure 4C-D). Meanwhile CHX treatment resulted in a significant decrease in TTC7B protein levels, indicating that GIPC1 silencing accelerates TTC7B degradation (Figure 4E-F). To confirm if GIPC1 affects the ubiquitination of TTC7B, ubiquitin and TTC7B were co-transfected into 293T cells. Overexpression of GIPC1 decreased the ubiquitination level of TTC7B, suggesting that GIPC1 inhibits TTC7B ubiquitination (Figure 4G-H). These findings demonstrate that GIPC1 interacts with and stabilizes TTC7B by inhibiting its ubiquitination.

Investigate the mechanism by which GIPC1 regulates TTC7B ubiquitination, E3 ubiquitin ligases bound to TTC7B were analyzed using Co-IP combined with protein profiling (Figure S4A and Table S6). Focusing on ligases with sequence coverage exceeding 5%, we found that TTC7B interacted with TRIM21 (Figure 4I). Moreover, TRIM21 knockdown resulted in upregulation of TTC7B expression (Figure 4J). Based on these findings, we then investigated whether GIPC1 regulates TRIM21 and found that GIPC1 negatively modulated TRIM21 and interacts with it (Figure 4K-L). Additional experiments supported these findings (Figure 4M). Cellular IF staining indicated that TTC7B and TRIM21 co-localize within cells, while GIPC1 is also found in close proximity in TRIM21 (Figure 4N), providing a spatial basis for their interaction. Based on these observations, we propose that GIPC1 may inhibit TTC7B ubiquitination by reducing the expression of the E3 ubiquitin ligase TRIM21, thereby contributing to the stabilization of TTC7B protein levels.

To explore the potential mechanism by which GIPC1 influences CRC, we divided tumor patients in the GSE32323 dataset into high-expression and low-expression groups based on GIPC1 expression. Then, we performed enrichment analysis on the differential genes in tumor tissues from these two groups. The results showed that the mTOR and NF-κB pathways were significantly enriched (Figure S4B-C). Further investigation showed that GIPC1 knockdown resulted in increased phosphorylation levels of mTOR and NF-κB, denoted as p-mTOR and p-NF-κB, respectively (Figure S4D). Collectively, these results indicate that GIPC1 promotes the stabilization of TTC7B and inhibits the mTOR/NF-κB signaling pathway in CRC.

### TTC7B inhibits chemoresistance, proliferation, and metastasis

**Figure 5:**
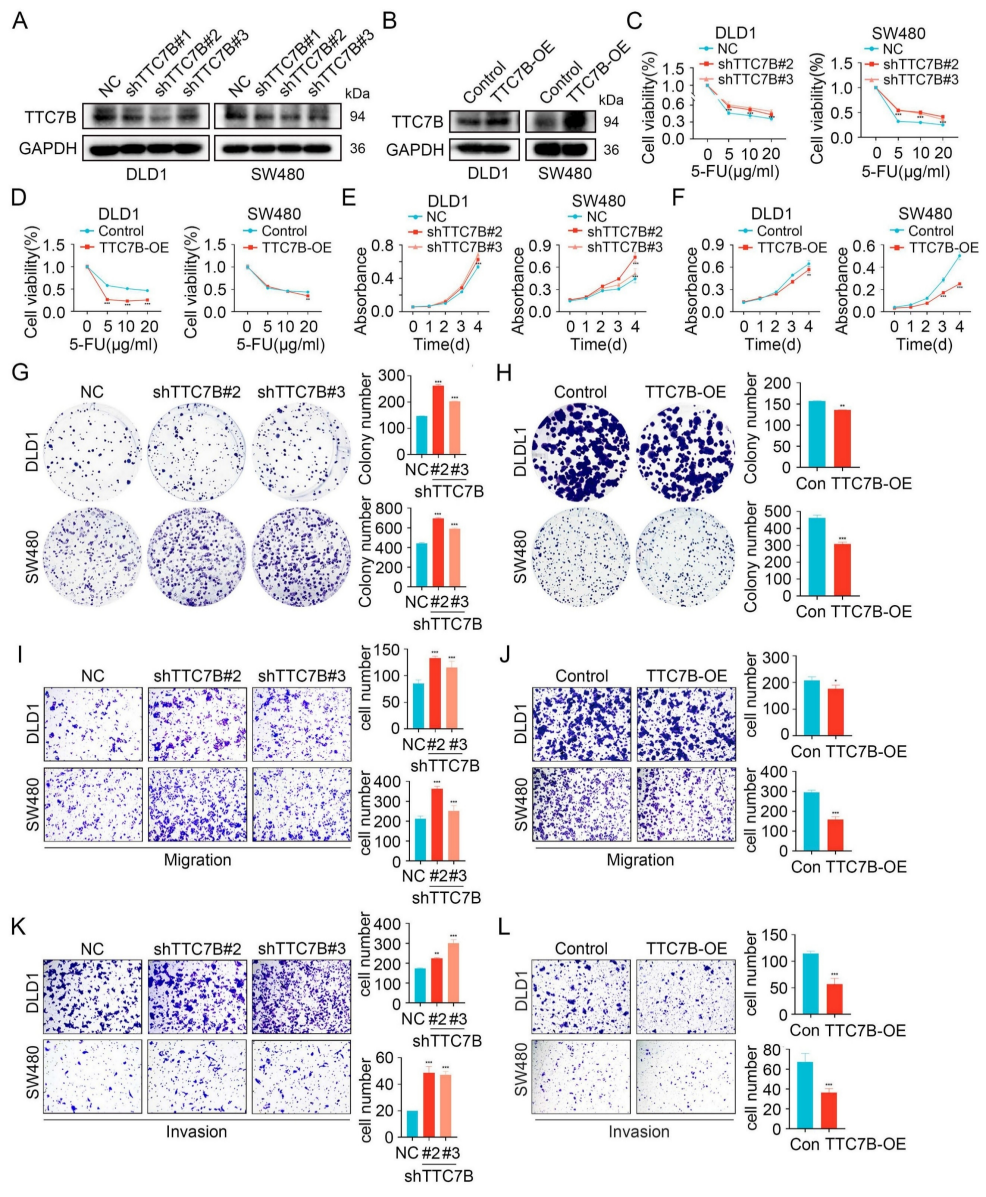
TTC7B inhibits chemoresistance, proliferation, and metastasis. (A) Knockdown of TTC7B using three independent shRNAs and the knockdown efficiency was examined. (B) Overexpression of TTC7B was detected. (C-D) Cell viability measured following exposure to varying concentrations of 5-FU. (E-H) CCK8 and colony formation assays show proliferation capacity. (I-L) Transwell assays evaluated the migratory and invasive capabilities. Data are presented as mean ± SD. *P < 0.05, **P < 0.01, ***P < 0.001.

Our investigation found that TTC7B expression is decreased in CRC (Figure S5A-C). Furthermore, Kaplan-Meier survival analysis shows a significant association between decreased TTC7B expression and shorter Relapse-Free Survival (RFS) in CRC patients with pathological stages T1 and T2 (Figure S5D). Moreover, ROC curve analysis demonstrates that TTC7B expression has diagnostic value for CRC, with an AUC of 0.794 (Figure S5E). These findings suggest that TTC7B may be a key factor inhibiting CRC progression. To investigate this, we overexpressed or knocked down TTC7B in CRC cells (Figure 5A-B and Figure S6A-B). After 48 hours of treatment with the chemotherapeutic agent 5-FU, cell viability was assessed, which revealed increased viability in TTC7B knockdown cells and decreased viability in TTC7B overexpressing cells (Figure 5C-D and Figure S6C). Similar trends of altered cell viability were observed following treatment with OXA and CPT-11 (Figure S7A-D). CCK8 and colony formation assays showed that TTC7B knockdown enhanced cell proliferation, whereas overexpression inhibited it (Figure 5E-H and Figure S6D-G). Transwell assays further demonstrated that TTC7B knockdown promoted cell migration and invasion, while overexpression suppressed these processes (Figure 5I-L and Figure S6H-I). Considering that GIPC1 inhibits the mTOR/NF-κB signaling pathway, we investigated whether TTC7B also regulates this pathway. The results indicated that TTC7B knockdown activated mTOR/NF-κB pathway (Figure S8A). These findings suggest that TTC7B inhibits tumor cell growth, metastasis and chemoresistance while downregulating the mTOR/NF-κB signaling pathway in CRC.

### GIPC1 inhibits proliferation, migration, and invasion by regulating TTC7B

**Figure 6:**
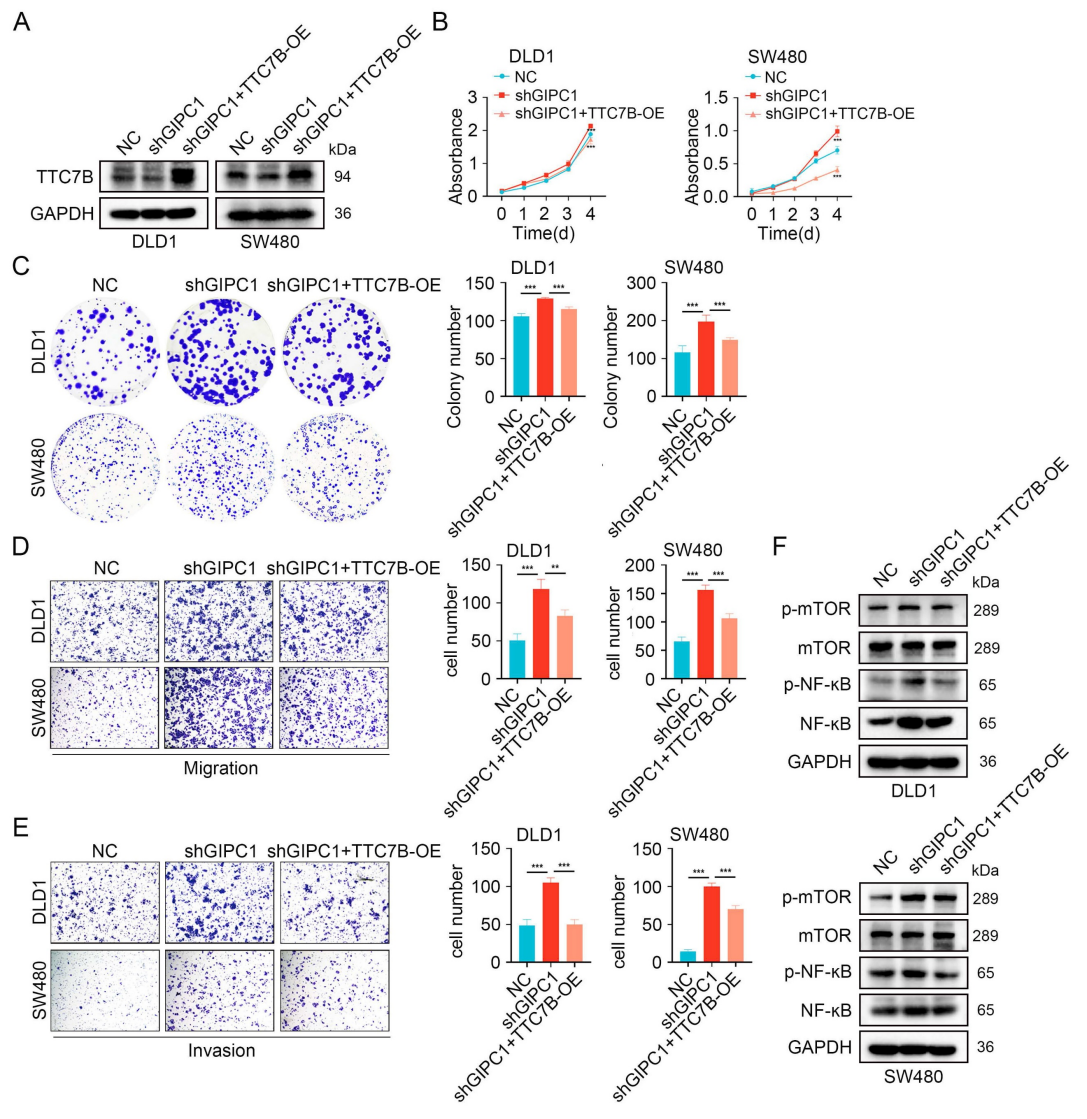
GIPC1 inhibits proliferation, migration, and invasion by regulating TTC7B. (A) Overexpression of TTC7B in GIPC1 knockdown DLD1 and SW480 cells. (B-C) CCK8 and colony formation assays assessed proliferation capability. (D-E) Transwell assays evaluated the migration and invasion capabilities of DLD1 and SW480 cells. (F) Overexpression of TTC7B in GIPC1 knockdown DLD1 and SW480 cells, followed by analysis of mTOR, NF-κB, and their phosphorylation levels. Data are presented as mean ± SD. **P < 0.01, ***P < 0.001.

We further examined how TTC7B is involved in GIPC1-mediated suppression of proliferation, migration, and invasion in CRC. We conducted rescue experiments in DLD1 and SW480 cells by stably knocking down GIPC1 and then overexpressing TTC7B (Figure 6A). GIPC1 knockdown led to increased proliferation and colony-forming ability in DLD1 and SW480 cells, effects partially reversed by TTC7B overexpression (Figure 6B-C). Moreover, TTC7B overexpression partially reversed migration and invasion induced by GIPC1 knockdown in these cells (Figure 6D-E). These results suggest that GIPC1 inhibits proliferation, migration, and invasion through the regulation of TTC7B.

Furthermore, stable GIPC1 knockdown increased activation mTOR/NF-κB pathway in DLD1 and SW480 cells, which was partially restored by TTC7B overexpression (Figure 6F).

### GIPC1 inhibits chemoresistance by regulating TTC7B in CRC

**Figure 7:**
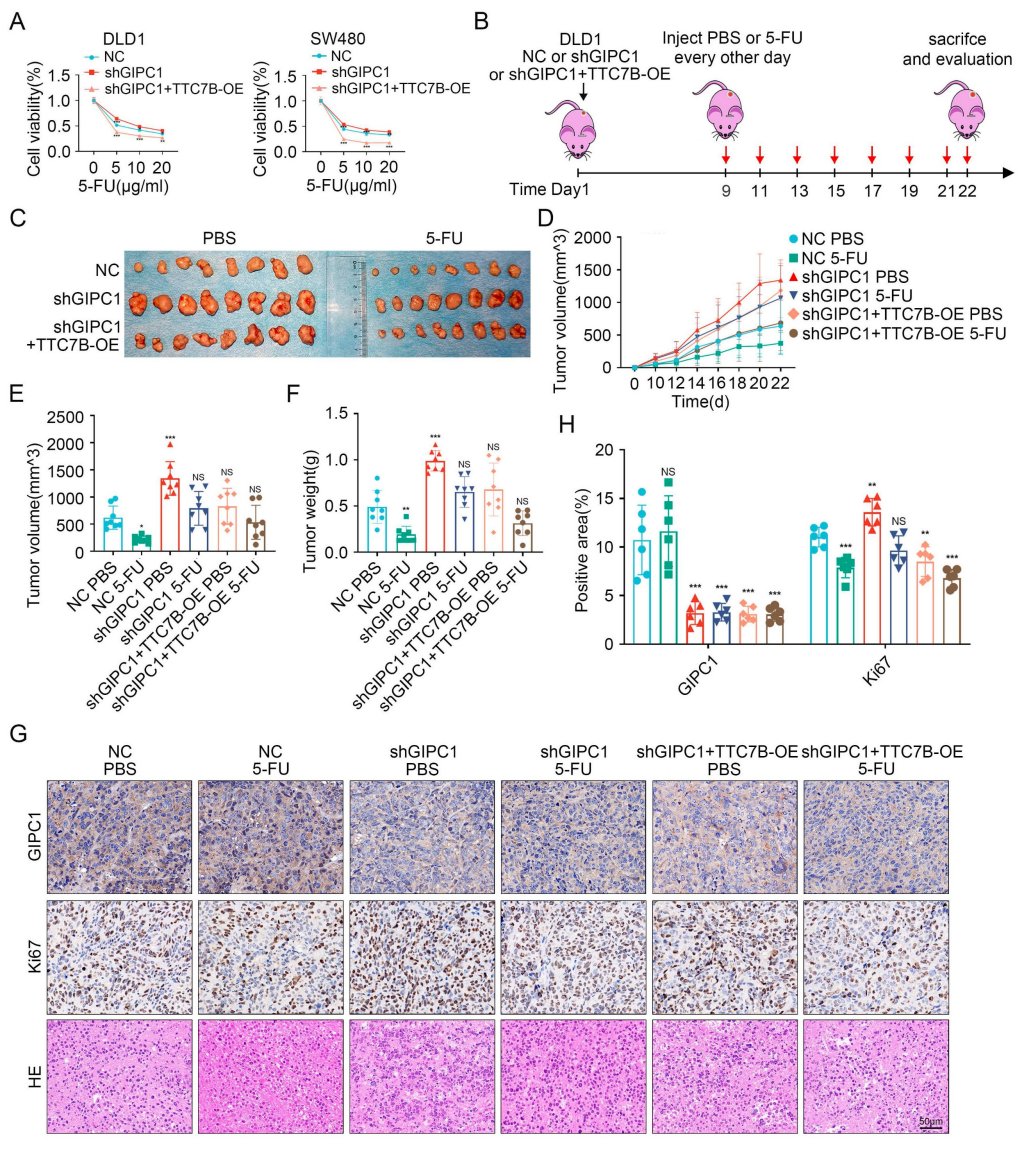
GIPC1 reverses chemoresistance through TTC7B in colorectal cancer. (A) Cell viability of DLD1 and SW480 cells after exposure to varying concentrations of 5-FU. (B) Schematic diagram of 5-FU treatment in a colorectal cancer xenograft animal model. (C) Representative images of neoplasms obtained from each mouse group (n = 8). (D-F) Tumor volume and weight measurements (n = 8). (G) H&E and IHC staining of tumor sections from various groups. Scale bar = 50 µm. (H) GIPC1 and Ki67 positive area in tumor sections from different groups. Data are presented as mean ± SD, *P < 0.05, **P < 0.01, ***P < 0.001, ns: not significant.

Given the association between GIPC1, TTC7B, and chemoresistance in CRC, we investigated the role of TTC7B in GIPC1-mediated chemoresistance. Rescue experiments were initially conducted in DLD1 and SW480 cells. Following treatment with 5-FU, OXA, or CPT-11, stable GIPC1 knockdown significantly increased cell viability, which was partially reversed by TTC7B overexpression (Figure 7A and Figure S9A-B). A xenograft tumor model was then established (Figure 7B). Tumor volume and weight were significantly reduced after 5-FU treatment in the control group. Compared to the shGIPC1 group, the shGIPC1+TTC7B-OE group exhibited slower tumor growth and lower tumor weight. Furthermore, in the shGIPC1+5-FU treatment group, TTC7B overexpression further suppressed tumor progression in DLD1 cells (Figure 7C-F). These* in vivo* results confirm that GIPC1 modulates CRC resistance to 5-FU through TTC7B. Ki67 and H&E staining assessed tumor proliferative. The results showed that 5-FU treatment reduced the proliferative of DLD1 cells in the control group. GIPC1 knockdown increased proliferation, which was reversed by TTC7B overexpression. Furthermore, the combination of shGIPC1+TTC7B-OE with 5-FU treatment further inhibited tumor growth (Figure 7G-H).

### LNP-loaded GIPC1 mRNA delivery reduces tumor burden in CDX-resistant model

**Figure 8:**
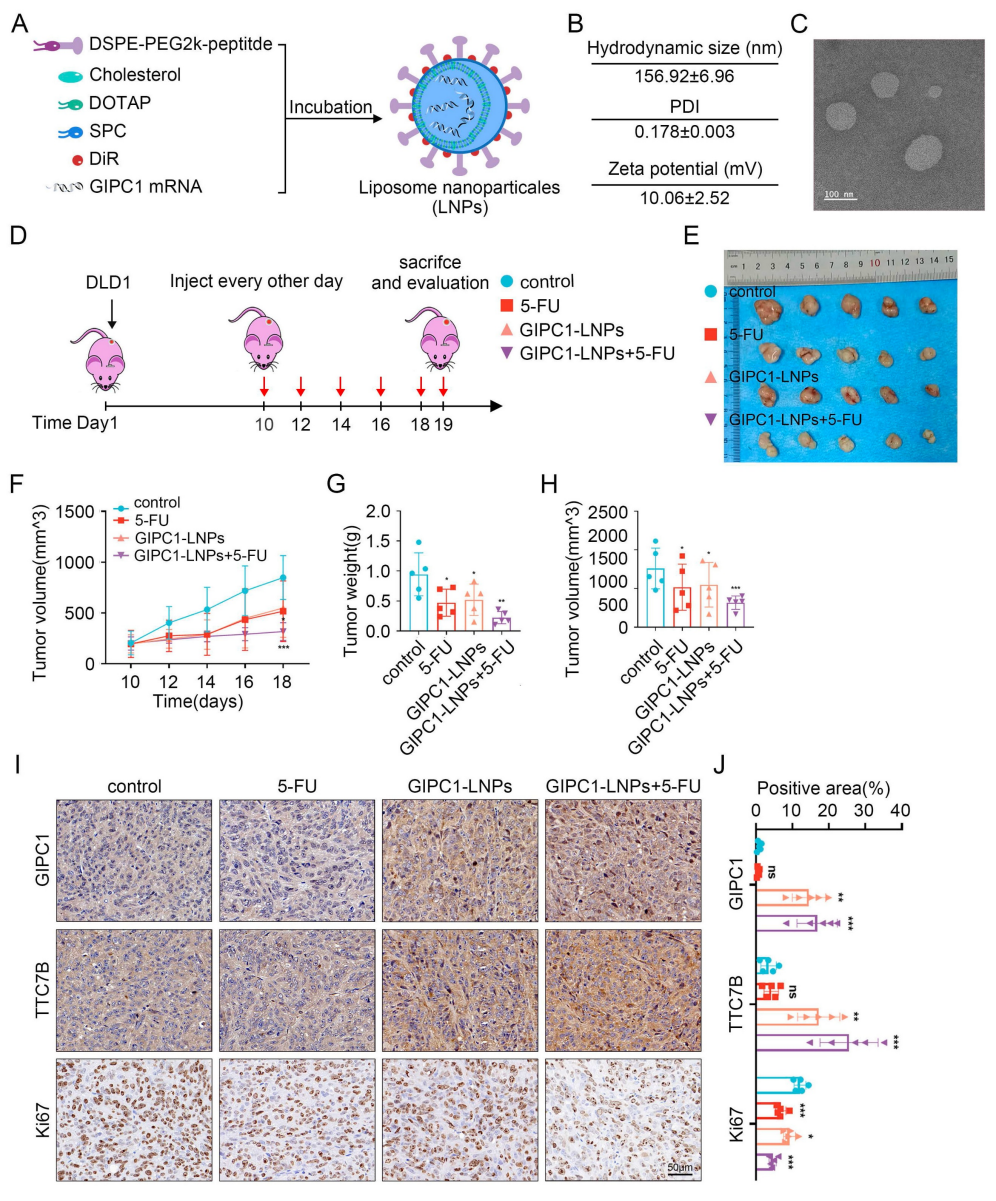
The antitumor function of GIPC1-LNPs in chemotherapy-resistant CDX models. (A) Schematic representation of the synthesis of lipid nanoparticles (LNPs) delivering GIPC1 mRNA. (B) Average dimensions, PDI, and zeta potential of GIPC1-LNPs. (C) TEM images of GIPC1-LNPs. Scale bar = 100 nm. (D) Schematic diagram of GIPC1-LNPs treatment in colorectal cancer CDX animal models. (E) Representative images of tumors from each mouse group (n = 5). (F-H) Tumor volume and weight measurements (n = 5). (I-J) IHC staining and GIPC1, TTC7B, and Ki67 positive area of tumor sections from various groups. Scale bar = 50 µm. Data are presented as mean ± SD. *P < 0.05, **P < 0.01, ***P < 0.001. ns: not significant.

In view of GIPC1's role in inhibiting CRC growth, restoring GIPC1 expression was hypothesized as a potential strategy for CRC treatment. To enhance CRC therapy, GIPC1 mRNA was synthesized (Figure S10A-B), and targeting peptides along with DIR were displayed on the surface of liposomes encapsulating the mRNA. These modifications resulted in liposomal nanoparticles (GIPC1-LNPs) designed to overexpress GIPC1 (Figure 8A). The hydrodynamic diameter of GIPC1-LNPs was 156.92±6.96 nm, with a PDI of 0.178±0.003 and a zeta potential of 10.06±2.52 mV (Figure 8B and Figure S11A-C). TEM images revealed that GIPC1-LNPs exhibited a spherical structure (Figure 8C). The encapsulation efficiency of GIPC1-LNPs was 98.3%, and each milligram contained 37 μg of mRNA. Previous research by Dania et al. demonstrated that peptide-modified LNPs effectively targeted CRC cells 24. To confirm the targeting ability, DLD1 and SW480 cells were treated with DIR-labeled LNPs, and fluorescence imaging confirmed that the LNPs entered and accumulated in CRC cells (Figure S12A). Tumor targeting ability was then assessed *in vivo*. Upon intravenous injection into CDX model mice, fluorescence signals from LNPs primarily accumulated in subcutaneous tumors (Figure S12B). These results suggest that targeting peptide-modified LNPs loaded with mRNA effectively target tumor cells in preclinical colorectal tumor models.

To determine whether GIPC1 mRNA inhibits tumor formation *in vivo*, we constructed a preclinical CDX-chemoresistance model (Figure 8D). Tumor size was significantly reduced in the group treated with GIPC1-LNPs compared to the control group treated with carrier LNPs (Figure 8E-H). Moreover, the combination of GIPC1-LNPs with 5-FU resulted in further tumor shrinkage (Figure 8E-H). Histological analysis revealed increased protein levels of GIPC1 and TTC7B in tumors treated with GIPC1-LNPs, while the Ki67 proliferation index was significantly elevated in the control group, especially the combination therapy of GIPC1-LNPs and 5-FU notably inhibited tumor proliferation (Figure 8I-J). No pathological damage was observed in the heart, lungs, liver, kidneys, or spleen of CDX model mice (Figure S13A). The liposomal delivery system developed in this study efficiently delivers mRNA to tumor sites in clinically relevant CDX models. GIPC1-LNPs enhanced therapeutic efficacy against CRC in chemotherapy-resistant models.

## Discussion

Colorectal cancer (CRC) ranks as the third most commonly diagnosed cancer globally and the second leading cause of cancer-related deaths, with its incidence continuing to rise 25. 5-Fluorouracil (5-FU) is acknowledged as a primary chemotherapeutic drug for treating CRC. However, clinical evidence shows that a considerable proportion of CRC patients develop resistance to 5-FU after a period of therapy, resulting in tumor progression and therapeutic failure. This underscores the imperative to examine the fundamental mechanisms behind 5-FU resistance and CRC progression. Such research can offer valuable theoretical insights to improve CRC treatment strategies.

Recent studies have shown that the mechanisms of 5-FU resistance involve metabolic regulation, signaling pathway reprogramming, and the interaction of the tumor microenvironments 26-30. In this study, GIPC1 levels were significantly lower in CRC tissues than in adjacent non-malignant tissues, and its reduced expression correlated with poor prognosis in patients at pathological stages T1 and T2. Altered GIPC1 expression has been observed in multiple cancers, where it plays a critical role in tumor initiation, progression, and metastasis 13,31-35. GIPC1 has dual functions in the progression and metastasis of MACC1-driven primary colorectal cancer 14. The E6/E7 protein of HPV-18 may promote GIPC1 degradation *via* ubiquitination. This mechanism could significantly disrupt TGF-β signaling, ultimately causing acquired resistance to the pathway 15. In pancreatic cancer cells, GIPC facilitates vesicular transport or membrane stabilization of ABCG2, which promotes ABCG2 release from intracellular vesicles. This process mediates the efflux of therapeutic agents, ultimately leading to resistance against gemcitabine in cancer cells 16. However, the mechanisms of GIPC1-mediated regulation in CRC progression and chemoresistance remain unclear. To explore this, we investigated the role of GIPC1 in these processes. Our initial findings confirm that GIPC1 suppresses tumor growth and decreases chemoresistance in CRC. Specifically, CRC cells lacking GIPC1 exhibited increased proliferation, migration, invasion, and resistance to chemotherapy. Further investigation revealed that reduced levels of GIPC1 correlated with increased expression of phosphorylated mTOR (p-mTOR) and phosphorylated NF-κB (p-NF-κB) in CRC cells. These results highlight GIPC1's critical function in suppressing CRC progression and chemoresistance, primarily through inhibiting the mTOR/NF-κB signaling pathway. This finding positions GIPC1 as a potential tumor suppressor in CRC, providing new insights into its role in cancer biology.

We used Co-IP and mass spectrometry to identify proteins that interact with GIPC1, aiming to clarify how GIPC1 restrains CRC progression and chemotherapy resistance. Knockdown of GIPC1 induced downregulation of TTC7B expression. Co-IP and IF assays revealed that GIPC1 co-localizes and interacts with TTC7B. Follow-up experiments confirmed that a specific domain of GIPC1 mediates interacting with TTC7B, but the exact binding site crucial for their association remains unknown. The exact molecular mechanisms underlying GIPC1-mediated expression of TTC7B are not yet fully understood. We then used Co-IP combined with mass spectrometry to identify the E3 ubiquitin ligase regulating TTC7B protein, discovering TRIM21. In subsequent experiments, we found that TRIM21 suppressed TTC7B expression, and GIPC1 reduced TRIM21 expression. We also confirmed the interaction and co-localization of TRIM21, GIPC1, and TTC7B in cells. Based on these findings, we hypothesized that GIPC1 reduced the expression of TRIM21, thereby inhibiting TTC7B ubiquitination and maintaining TTC7B levels in CRC cells.

In this study, we found that GIPC1 reduced TTC7B ubiquitination by decreasing the expression of TRIM21, which helped maintain TTC7B protein expression and inhibited the mTOR/NF-κB signaling pathway in CRC cells, thereby suppressing CRC progression and chemotherapy resistance. This finding reveals the critical role of GIPC1 in these processes and highlights its potential as a therapeutic target for CRC. To explore this potential, we employed targeting peptide-modified lipid nanoparticles (LNPs) to specifically deliver GIPC1 mRNA to tumor cells. The data demonstrated that liposomes loaded with GIPC1 mRNA effectively targeted CRC tumors and inhibited tumor progression in the cell-derived xenograft (CDX) model. Additionally, the combined treatment of GIPC1-LNPs with 5-FU significantly inhibited the growth of CRC tumors. These results support the link between reduced GIPC1 levels and CRC progression and enhanced chemotherapy resistance.

Despite significant progress in understanding CRC chemoresistance, tumor progression, and therapeutic potential 36-40, several limitations remain. First, mRNA delivery offers the advantage of bypassing nuclear transport and transcription requirements. However, issues related to stability, targeting and security still persist 41-43. While LNPs modified with targeting peptides effectively deliver GIPC1 mRNA to tumor cells in CRC CDX models, its distribution in healthy tissues remains unavoidable. Therefore, exploring precision medicine approaches is essential to achieve optimal therapeutic outcomes and minimizing off-target effects. Additionally, the limited sample size and absence of multicenter clinical validation restrict the generalizability and applicability of the findings. Secondly, future research should include a larger, more diverse sample and a broader range of CRC subtypes to better analyze heterogeneity and comprehensively assess the therapeutic potential of GIPC1 in different disease forms.

## Conclusion

In summary, this study revealed the suppressive role of GIPC1 in CRC progression and chemotherapy resistance. The specific mechanism is that GIPC1 prevents ubiquitination and maintains the stability of TTC7B protein by downregulating the expression of E3 ubiquitin ligase TRIM21. This action inhibits the mTOR/NF-κB signaling pathway, which in turn slows down CRC progression and chemoresistance. Furthermore, the combination of GIPC1-LNPs with 5-FU significantly improves therapeutic efficacy of CRC. These findings suggest that GIPC1 could serve as a promising therapeutic target to improve prognosis and guide innovative treatment strategies for CRC patients.

## Supplementary Material

Supplementary figures and tables.
