# Supplementary material for: GIPC1 Restrains the Progression and Chemoresistance of Colorectal Cancer by Regulating TTC7B/mTOR/NF-κB Axis
Source: Int J Biol Sci. 2026 Jan 1;22(2):786–801. doi: 10.7150/ijbs.119064 (PMC12781073; doi:10.7150/ijbs.119064)
Supplement: Supplementary file 1 — Supplementary figures and tables. [file ijbsv22p0786s1.pdf]

Supporting Information

Table S1 Relative information of databases used in this study.

Source	Dataset names	RNA sequence	Platforms	Country	Last update date
GEO	GSE25070	HumanRef-8 v3.0 expression beadchip	GPL6883	USA	2017
GEO	GSE32323	Human Genome U133 Plus 2.0 Array	GPL570	Japan	2019
GEO	GSE113513	Human Gene Expression Array	GPL15207	China	2022
GEO	GSE54986	HumanHT-12 V4.0 expression beadchip	GPL10558	China	2018
GEO	GSE181722	NextSeq 500	GPL18573	Bulgaria	2021
TCGA	COAD-US	RNA sequence	Illumina	USA	-
TCGA	READ-US	RNA-sequence	Illumina	USA	-

Table S2 Knockdown shRNA sequences used in this study.

TRC Number	Sequence
GIPC1-shRNA	Sh1: CCGGCGACATGATCGAGGCCATTAACTCGAGTTAATGGCCTCGATCATGTCGTTTTTG<br>Sh2: CCGGACCAACGTCAAGGAGCTGTATCTCGAGATACAGCTCCTTGACGTTGGTTTTTC<br>Sh3: CCGGACCAACGTCAAGGAGCTGTATCTCGAGATACAGCTCCTTGACGTTGGTTTTTC
sTTC7B-shRNA	sh1: CCGGGTTTGGAGAAGCTGCCTATTTCTCGAGAAATAGGCAGCTTCTCCAAACTTTTTG<br>sh2: CCGGCGCTACCAAAGGACTTTGTTTCTCGAGAAACAAAGTCCTTTGGTAGCGTITTTG<br>sh3: CCGGCCTACAAGAATCCAATCTGATCTCGAGATCAGATTGGATTCTTGTAGGTTTTTG

4 **Table S3 Overexpressed RNA used in this study.**

Primer Name	Vector
GIPC1	pCDH-CMV-EF1A-T2A
TTC7B	pCDH-CMV-EF1A-T2A
GIPC1 Flag	pCMV-MCS-3*Flag
TTC7B-GFP	pEGFP-N1
GIPC1-GST	pGEX-4T-1
GIPC1 1-333	pCMV-MCS-3*Flag
GIPC1 137-333	pCMV-MCS-3*Flag
GIPC1 218-333	pCMV-MCS-3*Flag

5 **Table S4 Different antibodies used in this study.**

Antibody name	Sources
GIPC1 (WB, IHC, IF)	Proteintech, 14822-1-AP
TTC7B (WB, ELISA)	Proteintech, 25713-1-AP
TTC7B (WB, IP)	Abcam, ab19336
p-mTOR (WB, IHC, IF)	Proteintech, 67778-1-Ig
mTOR (WB, IHC, IF, IP, PLA)	Proteintech, 66888-1-Ig
p-NF-κB p65(WB, W-S, IP, IF, FC-FP)	CST, 3033S
NF-κB p65 (WB, IHC, IF, IP, CoIP, ChIP, ELISA)	Proteintech, 10745-1-AP
GFP Tag (WB, IHC, IF, IP, CoIP, ChIP, RIP, ELISA)	Proteintech, 66002-1-Ig
DDDDK-Tag (WB, IF/ICC, IP, FC, ELISA)	Abclonal, AE005
ubiquitin (WB, IP, CoIP)	Proteintech, 80992-1-RR
GST Tag (WB, IF, IP, CoIP, ChIP)	Proteintech, 10000-0-AP
TRIM21 (WB, IP, IHC, IF)	Proteintech, 12108-1-AP
TRIM4 (WB, IF, ELISA)	ABclonal, A15922
NOSIP (WB, ELISA)	ABclonal, A10024
HACE1 (WB, IHC, ELISA)	ABclonal, A9593
GAPDH (WB, IHC, IF, IP, CoIP, ELISA)	Proteintech, 60004-1-Ig

Table S5 Related to Figure 3; GIPC1 interacting proteins.

Gene-name	Description	Unique peptides	Sequence coverage [%]	Score
TTC7B	tr|H0YK02|H0YK02_HUMAN Tetratricopeptide repeat protein 7B (Fragment) OS=Homo sapiens OX=9606 GN=TTC7B PE=1 SV=1	28	39.4	323.31
DDX3X	tr|A0A0D9SF53|A0A0D9SF53_HUMAN ATP-dependent RNA helicase DDX3X OS=Homo sapiens OX=9606 GN=DDX3X PE=1 SV=1	4	38.1	28.523
ARI1A	sp|O14497|ARI1A_HUMAN AT-rich interactive domain-containing protein 1A OS=Homo sapiens OX=9606 GN=ARID1A PE=1 SV=3	1	37.2	5.8866
HNRNPDL	tr|A0A087WUK2|A0A087WUK2_HUMAN Heterogeneous nuclear ribonucleoprotein D-like OS=Homo sapiens OX=9606 GN=HNRNPDL PE=1 SV=1	8	35.1	70.682
TRNK1	sp|O15050|TRNK1_HUMAN TPR and ankyrin repeat-containing protein 1 OS=Homo sapiens OX=9606 GN=TRANK1 PE=2 SV=4	3	34.7	5.6006
EIF3D	tr|B0QYA4|B0QYA4_HUMAN Eukaryotic translation initiation factor 3 subunit D (Fragment) OS=Homo sapiens OX=9606 GN=EIF3D PE=1 SV=2	1	34.5	5.7428
TNRC18	tr|H9KVB4|H9KVB4_HUMAN Trinucleotide repeat-containing gene 18 protein OS=Homo sapiens OX=9606 GN=TNRC18 PE=1 SV=1	1	33.3	5.7639
MEFV	tr|F5GZV9|F5GZV9_HUMAN Pyrin OS=Homo sapiens OX=9606 GN=MEFV PE=1 SV=1	2	31.1	5.61
CYB5B	tr|D6RFH4|D6RFH4_HUMAN Cytochrome b5 type B OS=Homo sapiens OX=9606 GN=CYB5B PE=1 SV=1	1	31	5.6885
ACTN1	tr|H7C5W8|H7C5W8_HUMAN Alpha-actinin-1 (Fragment) OS=Homo sapiens OX=9606 GN=ACTN1 PE=1 SV=1	1	30.6	5.6482
ST18	tr|E5RK88|E5RK88_HUMAN Suppression	3	30	5.9447

	of tumorigenicity 18 protein (Fragment) OS=Homo sapiens OX=9606 GN=ST18 PE=4 SV=8			
ZC3H1	sp|O60293|ZC3H1_HUMAN Zinc finger C3H1 domain-containing protein OS=Homo sapiens OX=9606 GN=ZFC3H1 PE=1 SV=3	2	29.2	5.6025
HNRPQ	sp|O60506|HNRPQ_HUMAN Heterogeneous nuclear ribonucleoprotein Q OS=Homo sapiens OX=9606 GN=SYNCRIP PE=1 SV=2	6	29	37.635
UGDH	sp|O60701|UGDH_HUMAN UDP-glucose 6-dehydrogenase OS=Homo sapiens OX=9606 GN=UGDH PE=1 SV=1	2	28.2	7.3464
CTNND1	tr|C9JZR2|C9JZR2_HUMAN Catenin delta-1 OS=Homo sapiens OX=9606 GN=CTNND1 PE=1 SV=2	1	28.1	5.7564
H2BC15	tr|U3KQK0|U3KQK0_HUMAN Histone H2B OS=Homo sapiens OX=9606 GN=H2BC15 PE=1 SV=1	4	26.7	11.226
ERLN1	sp|O75477|ERLN1_HUMAN Erlin-1 OS=Homo sapiens OX=9606 GN=ERLIN1 PE=1 SV=2	1	25.9	5.7154
BANF1	sp|O75531|BAF_HUMAN Barrier-to-autointegration factor OS=Homo sapiens OX=9606 GN=BANF1 PE=1 SV=1	1	25.8	5.7861
ERLIN2	tr|E5RHW4|E5RHW4_HUMAN Erlin-2 (Fragment) OS=Homo sapiens OX=9606 GN=ERLIN2 PE=1 SV=1	1	25.1	20.297
VAPB	tr|E5RK64|E5RK64_HUMAN Vesicle-associated membrane protein-associated protein B/C OS=Homo sapiens OX=9606 GN=VAPB PE=1 SV=1	2	24.6	5.6493
AHSA1	tr|H0YJ63|H0YJ63_HUMAN Activator of 90 kDa heat shock protein ATPase homolog 1 (Fragment) OS=Homo sapiens OX=9606 GN=AHSA1 PE=1 SV=1	2	24.5	5.7612
LDHA	sp|P00338|LDHA_HUMAN L-lactate dehydrogenase A chain OS=Homo sapiens OX=9606 GN=LDHA PE=1 SV=2	13	24.4	32.551
AATM	sp|P00505|AATM_HUMAN Aspartate aminotransferase, mitochondrial OS=Homo sapiens OX=9606 GN=GOT2 PE=1 SV=3	1	23.9	5.8027
KAD1	sp|P00568|KAD1_HUMAN Adenylate kinase isoenzyme 1 OS=Homo sapiens	3	23.8	6.8514

	OX=9606 GN=AK1 PE=1 SV=3			
LV151	sp|P01701|LV151_HUMAN Immunoglobulin lambda variable 1-51 OS=Homo sapiens OX=9606 GN=IGLV1-51 PE=1 SV=2	2	22.5	6.1023
LV208	sp|P01709|LV208_HUMAN Immunoglobulin lambda variable 2-8 OS=Homo sapiens OX=9606 GN=IGLV2-8 PE=1 SV=2	3	22.4	6.988
IGHG1	tr|A0A0A0MS07|A0A0A0MS07_HUMAN Immunoglobulin heavy constant gamma 1 (Fragment) OS=Homo sapiens OX=9606 GN=IGHG1 PE=1 SV=1	2	21.6	60.233
HBB	tr|F8W6P5|F8W6P5_HUMAN Hemoglobin subunit beta (Fragment) OS=Homo sapiens OX=9606 GN=HBB PE=1 SV=1	3	21.6	8.3481
COL1A1	sp|P02452|CO1A1_HUMAN Collagen alpha-1(I) chain OS=Homo sapiens OX=9606 GN=COL1A1 PE=1 SV=5	7	21.6	125.53
KRT14	sp|P02533|K1C14_HUMAN Keratin, type I cytoskeletal 14 OS=Homo sapiens OX=9606 GN=KRT14 PE=1 SV=4	6	21.4	313.87
KRT6C	sp|P48668|K2C6C_HUMAN Keratin, type II cytoskeletal 6C OS=Homo sapiens OX=9606 GN=KRT6C PE=1 SV=3	0	21.3	18.524
LMNA	sp|P02545|LMNA_HUMAN Prelamin-A/C OS=Homo sapiens OX=9606 GN=LMNA PE=1 SV=1	5	21	26.374
ALB	tr|H0YA55|H0YA55_HUMAN Serum albumin (Fragment) OS=Homo sapiens OX=9606 GN=ALB PE=1 SV=1	22	21	112.75
GBA	tr|A0A0G2JNZ5|A0A0G2JNZ5_HUMAN Glucosylceramidase OS=Homo sapiens OX=9606 GN=GBA PE=1 SV=1	3	20.4	9.3831
ALDOA	tr|H3BQN4|H3BQN4_HUMAN Fructose-bisphosphate aldolase OS=Homo sapiens OX=9606 GN=ALDOA PE=1 SV=1	8	20.2	49.188
ANXA1	sp|P04083|ANXA1_HUMAN Annexin A1 OS=Homo sapiens OX=9606 GN=ANXA1 PE=1 SV=2	7	19.7	35.767
KRT6B	sp|P04259|K2C6B_HUMAN Keratin, type II cytoskeletal 6B OS=Homo sapiens OX=9606 GN=KRT6B PE=1 SV=5	0	19.7	14.829
KRT1	sp|P04264|K2C1_HUMAN Keratin, type II	64	19.1	323.31

	cytoskeletal 1 OS=Homo sapiens OX=9606 GN=KRT1 PE=1 SV=6			
GAPDH	tr|E7EUT5|E7EUT5_HUMAN Glyceraldehyde-3-phosphate dehydrogenase OS=Homo sapiens OX=9606 GN=GAPDH PE=1 SV=1	13	19.1	41.068
RPN1	tr|B7Z4L4|B7Z4L4_HUMAN Dolichyl-diphosphooligosaccharide--protein glycosyltransferase subunit 1 OS=Homo sapiens OX=9606 GN=RPN1 PE=1 SV=1	4	19	27.464
RPN2	tr|F2Z3K5|F2Z3K5_HUMAN Dolichyl-diphosphooligosaccharide--protein glycosyltransferase subunit 2 (Fragment) OS=Homo sapiens OX=9606 GN=RPN2 PE=1 SV=1	1	19	6.6927
SLC25A5	sp|P05141|ADT2_HUMAN ADP/ATP translocase 2 OS=Homo sapiens OX=9606 GN=SLC25A5 PE=1 SV=7	2	19	12.867
RPLP1	sp|P05386|RLA1_HUMAN 60S acidic ribosomal protein P1 OS=Homo sapiens OX=9606 GN=RPLP1 PE=1 SV=1	1	18.6	6.0888
RPLP0	tr|F8VWS0|F8VWS0_HUMAN 60S acidic ribosomal protein P0 OS=Homo sapiens OX=9606 GN=RPLP0 PE=1 SV=1	5	18.3	34.396
KRT18	tr|F8VZY9|F8VZY9_HUMAN Keratin, type I cytoskeletal 18 OS=Homo sapiens OX=9606 GN=KRT18 PE=1 SV=1	11	17.5	59.312
K2C8	sp|P05787|K2C8_HUMAN Keratin, type II cytoskeletal 8 OS=Homo sapiens OX=9606 GN=KRT8 PE=1 SV=7	16	17.4	201.5
IGKV4-1	sp|P06312|KV401_HUMAN Immunoglobulin kappa variable 4-1 OS=Homo sapiens OX=9606 GN=IGKV4-1 PE=1 SV=1	3	16.9	8.6033
ATP5F1B	sp|P06576|ATPB_HUMAN ATP synthase subunit beta, mitochondrial OS=Homo sapiens OX=9606 GN=ATP5F1B PE=1 SV=3	11	16.1	47.521
ENO1	sp|P06733|ENOA_HUMAN Alpha-enolase OS=Homo sapiens OX=9606 GN=ENO1 PE=1 SV=2	16	16	68.757
GPI	tr|A0A0J9YXP8|A0A0J9YXP8_HUMAN Glucose-6-phosphate isomerase (Fragment) OS=Homo sapiens OX=9606 GN=GPI PE=1	1	15.6	6.5696

	SV=1			
P4HB	sp|P07237|PDIA1_HUMAN Protein disulfide-isomerase OS=Homo sapiens OX=9606 GN=P4HB PE=1 SV=3	4	15.4	42.559
ANXA2	sp|P07355|ANXA2_HUMAN Annexin A2 OS=Homo sapiens OX=9606 GN=ANXA2 PE=1 SV=2	8	15.2	54.663
TUBB	tr|Q5ST81|Q5ST81_HUMAN Tubulin beta chain OS=Homo sapiens OX=9606 GN=TUBB PE=1 SV=1	2	15.1	8.8247
PFN1	tr|K7EJ44|K7EJ44_HUMAN Profilin OS=Homo sapiens OX=9606 GN=PFN1 PE=1 SV=1	2	15	5.6155
HSP90AA1	sp|P07900|HS90A_HUMAN Heat shock protein HSP 90-alpha OS=Homo sapiens OX=9606 GN=HSP90AA1 PE=1 SV=5	7	14.8	106.68
HNRNPC	tr|G3V555|G3V555_HUMAN Heterogeneous nuclear ribonucleoproteins C1/C2 (Fragment) OS=Homo sapiens OX=9606 GN=HNRNPC PE=1 SV=1	1	14.7	5.5968
COL1A2	tr|A0A087WTA8|A0A087WTA8_HUMAN Collagen alpha-2(I) chain OS=Homo sapiens OX=9606 GN=COL1A2 PE=1 SV=1	3	14.4	21.767
HSP90AB1	sp|P08238|HS90B_HUMAN Heat shock protein HSP 90-beta OS=Homo sapiens OX=9606 GN=HSP90AB1 PE=1 SV=4	6	14.3	72.661
CYC1	sp|P08574|CY1_HUMAN Cytochrome c1, heme protein, mitochondrial OS=Homo sapiens OX=9606 GN=CYC1 PE=1 SV=3	1	13.9	5.769
KRT19	sp|P08727|K1C19_HUMAN Keratin, type I cytoskeletal 19 OS=Homo sapiens OX=9606 GN=KRT19 PE=1 SV=4	3	13.9	47.814
ANXA5	tr|E9PHT9|E9PHT9_HUMAN Annexin OS=Homo sapiens OX=9606 GN=ANXA5 PE=1 SV=1	1	13.7	6.5096
KRT16	sp|P08779|K1C16_HUMAN Keratin, type I cytoskeletal 16 OS=Homo sapiens OX=9606 GN=KRT16 PE=1 SV=4	6	13.5	19.837
RPSA	tr|C9J9K3|C9J9K3_HUMAN 40S ribosomal protein SA (Fragment) OS=Homo sapiens OX=9606 GN=RPSA PE=1 SV=8	4	13.5	32.366
HNRNPA1	tr|F8VTQ5|F8VTQ5_HUMAN Heterogeneous nuclear ribonucleoprotein A1 (Fragment) OS=Homo sapiens OX=9606	2	13.3	6.0694

	GN=HNRNPA1 PE=1 SV=1			
GSTP1	tr|A0A2R8Y5E5|A0A2R8Y5E5_HUMAN Glutathione S-transferase P OS=Homo sapiens OX=9606 GN=GSTP1 PE=1 SV=1	1	13.3	22.172
PARP1	sp|P09874|PARP1_HUMAN Poly [ADP-ribose] polymerase 1 OS=Homo sapiens OX=9606 GN=PARP1 PE=1 SV=4	28	12.8	312.48
UBB	tr|J3QSA3|J3QSA3_HUMAN Polyubiquitin-B (Fragment) OS=Homo sapiens OX=9606 GN=UBB PE=1 SV=1	3	12.7	9.2399
HSPA1A	sp|P0DMV8|HS71A_HUMAN Heat shock 70 kDa protein 1A OS=Homo sapiens OX=9606 GN=HSPA1A PE=1 SV=1	0	12.7	11.122
HSPD1	sp|P10809|CH60_HUMAN 60 kDa heat shock protein, mitochondrial OS=Homo sapiens OX=9606 GN=HSPD1 PE=1 SV=2	11	12.6	106.5
HSPA5	sp|P11021|BIP_HUMAN Endoplasmic reticulum chaperone BiP OS=Homo sapiens OX=9606 GN=HSPA5 PE=1 SV=2	6	12.4	25.68
HSPA8	sp|P11142|HSP7C_HUMAN Heat shock cognate 71 kDa protein OS=Homo sapiens OX=9606 GN=HSPA8 PE=1 SV=1	13	12.2	266.39
DBT	tr|Q5VVL7|Q5VVL7_HUMAN Dihydrolipoamide acetyltransferase component of pyruvate dehydrogenase complex OS=Homo sapiens OX=9606 GN=DBT PE=1 SV=1	8	12.2	46.511
TOP1	sp|P11387|TOP1_HUMAN DNA topoisomerase 1 OS=Homo sapiens OX=9606 GN=TOP1 PE=1 SV=2	1	12.1	7.1249
PABPC1	sp|P11940|PABP1_HUMAN Polyadenylate-binding protein 1 OS=Homo sapiens OX=9606 GN=PABPC1 PE=1 SV=2	3	11.9	31.989
IMPDH2	sp|P12268|IMDH2_HUMAN Inosine-5-monophosphate dehydrogenase 2 OS=Homo sapiens OX=9606 GN=IMPDH2 PE=1 SV=2	2	11.6	14.044
PIP	sp|P12273|PIP_HUMAN Prolactin-inducible protein OS=Homo sapiens OX=9606 GN=PIP PE=1 SV=1	1	11.5	6.1008
CKB	sp|P12277|KCRB_HUMAN Creatine kinase B-type OS=Homo sapiens OX=9606 GN=CKB PE=1 SV=1	2	11.3	11.782
ANXA3	tr|D6RA82|D6RA82_HUMAN Annexin	2	11.3	6.0723

	OS=Homo sapiens OX=9606 GN=ANXA3 PE=1 SV=1			
XRCC6	sp|P12956|XRCC6_HUMAN X-ray repair cross-complementing protein 6 OS=Homo sapiens OX=9606 GN=XRCC6 PE=1 SV=2	42	11.1	323.31
XRCC5	sp|P13010|XRCC5_HUMAN X-ray repair cross-complementing protein 5 OS=Homo sapiens OX=9606 GN=XRCC5 PE=1 SV=3	28	11	241.01
EEF2	sp|P13639|EF2_HUMAN Elongation factor 2 OS=Homo sapiens OX=9606 GN=EEF2 PE=1 SV=4	1	10.9	5.8491
RNH1	tr|H0YCR7|H0YCR7_HUMAN Ribonuclease inhibitor (Fragment) OS=Homo sapiens OX=9606 GN=RNH1 PE=1 SV=1	8	10.9	37.58
KRT10	sp|P13645|K1C10_HUMAN Keratin, type I cytoskeletal 10 OS=Homo sapiens OX=9606 GN=KRT10 PE=1 SV=6	75	10.6	323.31
KRT5	sp|P13647|K2C5_HUMAN Keratin, type II cytoskeletal 5 OS=Homo sapiens OX=9606 GN=KRT5 PE=1 SV=3	16	10.6	59.801
PRKAR2A	tr|H7C1L0|H7C1L0_HUMAN cAMP-dependent protein kinase type II-alpha regulatory subunit (Fragment) OS=Homo sapiens OX=9606 GN=PRKAR2A PE=1 SV=1	1	10.5	5.8183
MTHFD2	tr|B9A062|B9A062_HUMAN Bifunctional methylenetetrahydrofolate dehydrogenase/cyclohydrolase, mitochondrial OS=Homo sapiens OX=9606 GN=MTHFD2 PE=1 SV=1	1	10.5	5.6548
PKM	sp|P14618|KPYM_HUMAN Pyruvate kinase PKM OS=Homo sapiens OX=9606 GN=PKM PE=1 SV=4	17	10.4	61.641
HSP90B1	sp|P14625|ENPL_HUMAN Endoplasmin OS=Homo sapiens OX=9606 GN=HSP90B1 PE=1 SV=1	7	10	32.579
JUP	sp|P14923|PLAK_HUMAN Junction plakoglobin OS=Homo sapiens OX=9606 GN=JUP PE=1 SV=3	4	10	13.089
PGAM2	sp|P15259|PGAM2_HUMAN Phosphoglycerate mutase 2 OS=Homo sapiens OX=9606 GN=PGAM2 PE=1 SV=3	2	9.8	5.9608
EZR	tr|E7EQR4|E7EQR4_HUMAN Ezrin	7	9.7	39.085

	OS=Homo sapiens OX=9606 GN=EZR PE=1 SV=3			
NME1	tr|C9K028|C9K028_HUMAN Nucleoside diphosphate kinase A (Fragment) OS=Homo sapiens OX=9606 GN=NME1 PE=1 SV=1	1	9.4	6.1383
RPS2	tr|E9PQD7|E9PQD7_HUMAN 40S ribosomal protein S2 OS=Homo sapiens OX=9606 GN=RPS2 PE=1 SV=1	3	9.1	13.533
H1-5	sp|P16401|H15_HUMAN Histone H1.5 OS=Homo sapiens OX=9606 GN=H1-5 PE=1 SV=3	2	8.8	6.0356
H1-2	sp|P16403|H12_HUMAN Histone H1.2 OS=Homo sapiens OX=9606 GN=H1-2 PE=1 SV=2	4	8.8	16.773
YBX3	sp|P16989|YBOX3_HUMAN Y-box-binding protein 3 OS=Homo sapiens OX=9606 GN=YBX3 PE=1 SV=4	2	8.6	5.9029
HSPA6	sp|P17066|HSP76_HUMAN Heat shock 70 kDa protein 6 OS=Homo sapiens OX=9606 GN=HSPA6 PE=1 SV=2	1	8.4	6.0694
DDX5	tr|J3KTA4|J3KTA4_HUMAN Probable ATP-dependent RNA helicase DDX5 OS=Homo sapiens OX=9606 GN=DDX5 PE=1 SV=1	9	8.2	44.517
TCP1	sp|P17987|TCPA_HUMAN T-complex protein 1 subunit alpha OS=Homo sapiens OX=9606 GN=TCP1 PE=1 SV=1	2	8.2	15.924
RPL7	sp|P18124|RL7_HUMAN 60S ribosomal protein L7 OS=Homo sapiens OX=9606 GN=RPL7 PE=1 SV=1	5	8.1	21.26
VCL	tr|A0A096LPE1|A0A096LPE1_HUMAN Vinculin OS=Homo sapiens OX=9606 GN=VCL PE=1 SV=1	2	8	6.0297
NCL	tr|H7BY16|H7BY16_HUMAN Nucleolin (Fragment) OS=Homo sapiens OX=9606 GN=NCL PE=1 SV=8	2	8	11.968
TRIM21	sp|P19474|RO52_HUMAN E3 ubiquitin-protein ligase TRIM21 OS=Homo sapiens OX=9606 GN=TRIM21 PE=1 SV=1	3	7.8	21.275
RAB6B	tr|C9JB90|C9JB90_HUMAN Ras-related protein Rab-6B (Fragment) OS=Homo sapiens OX=9606 GN=RAB6B PE=4 SV=1	3	7.7	5.6456
FLNA	tr|Q60FE5|Q60FE5_HUMAN Filamin-A OS=Homo sapiens OX=9606 GN=FLNA PE=1 SV=1	11	7.6	69.703

RPS4Y1	tr|C9JEH7|C9JEH7_HUMAN 40S ribosomal protein S4, Y isoform 1 (Fragment) OS=Homo sapiens OX=9606 GN=RPS4Y1 PE=1 SV=1	1	7.5	6.2875
SFPQ	sp|P23246|SFPQ_HUMAN Splicing factor, proline- and glutamine-rich OS=Homo sapiens OX=9606 GN=SFPQ PE=1 SV=2	6	7.4	38.763
HNRNPA2B1	sp|P22626|ROA2_HUMAN Heterogeneous nuclear ribonucleoproteins A2/B1 OS=Homo sapiens OX=9606 GN=HNRNPA2B1 PE=1 SV=2	10	7.4	79.184
RPS3	tr|F2Z2S8|F2Z2S8_HUMAN 40S ribosomal protein S3 OS=Homo sapiens OX=9606 GN=RPS3 PE=1 SV=1	4	7.3	21.21
AHCY	sp|P23526|SAHH_HUMAN Adenosylhomocysteinase OS=Homo sapiens OX=9606 GN=AHCY PE=1 SV=4	3	7.2	11.992
CFL1	tr|E9PS23|E9PS23_HUMAN Cofilin-1 (Fragment) OS=Homo sapiens OX=9606 GN=CFL1 PE=1 SV=8	3	7.1	13.359
EIF4B	tr|F8W0K0|F8W0K0_HUMAN Eukaryotic translation initiation factor 4B (Fragment) OS=Homo sapiens OX=9606 GN=EIF4B PE=1 SV=8	3	7.1	6.0948
MCM3	sp|P25205|MCM3_HUMAN DNA replication licensing factor MCM3 OS=Homo sapiens OX=9606 GN=MCM3 PE=1 SV=3	1	6.9	6.1778
ATP5F1A	sp|P25705|ATPA_HUMAN ATP synthase subunit alpha, mitochondrial OS=Homo sapiens OX=9606 GN=ATP5F1A PE=1 SV=1	5	6.8	11.543
AZGP1	sp|P25311|ZA2G_HUMAN Zinc-alpha-2-glycoprotein OS=Homo sapiens OX=9606 GN=AZGP1 PE=1 SV=2	15	6.8	73.224
RPL13	tr|J3QSB4|J3QSB4_HUMAN 60S ribosomal protein L13 (Fragment) OS=Homo sapiens OX=9606 GN=RPL13 PE=1 SV=1	4	6.8	12.177
PTBP1	tr|A0A087WU68|A0A087WU68_HUMAN Polypyrimidine tract-binding protein 1 (Fragment) OS=Homo sapiens OX=9606 GN=PTBP1 PE=1 SV=1	1	6.7	5.6788
EEF1G	sp|P26641|EF1G_HUMAN Elongation factor 1-gamma OS=Homo sapiens	5	6.7	25.084

	OX=9606 GN=EEF1G PE=1 SV=3			
RFA1	sp|P27694|RFA1_HUMAN Replication protein A 70 kDa DNA-binding subunit OS=Homo sapiens OX=9606 GN=RPA1 PE=1 SV=2	3	6.6	8.2416
CALR	sp|P27797|CALR_HUMAN Calreticulin OS=Homo sapiens OX=9606 GN=CALR PE=1 SV=1	5	6.6	18.074
CANX	tr|D6RHJ3|D6RHJ3_HUMAN Calnexin (Fragment) OS=Homo sapiens OX=9606 GN=CANX PE=1 SV=8	1	6.5	16.303
HTR2A	tr|A0A087WZJ9|A0A087WZJ9_HUMAN 5-hydroxytryptamine receptor 2A (Fragment) OS=Homo sapiens OX=9606 GN=HTR2A PE=4 SV=1	1	6.4	5.5867
TKT	sp|P29401|TKT_HUMAN Transketolase OS=Homo sapiens OX=9606 GN=TKT PE=1 SV=3	3	6.2	20.522
PRDX6	sp|P30041|PRDX6_HUMAN Peroxiredoxin-6 OS=Homo sapiens OX=9606 GN=PRDX6 PE=1 SV=3	1	6.2	5.6732
PRDX3	sp|P30048|PRDX3_HUMAN Thioredoxin-dependent peroxide reductase, mitochondrial OS=Homo sapiens OX=9606 GN=PRDX3 PE=1 SV=3	3	6	13.157
RPL12	sp|P30050|RL12_HUMAN 60S ribosomal protein L12 OS=Homo sapiens OX=9606 GN=RPL12 PE=1 SV=1	2	6	13.128
PDIA3	sp|P30101|PDIA3_HUMAN Protein disulfide-isomerase A3 OS=Homo sapiens OX=9606 GN=PDIA3 PE=1 SV=4	1	5.9	6.6557
HNRNPH3	sp|P31942|HNRH3_HUMAN Heterogeneous nuclear ribonucleoprotein H3 OS=Homo sapiens OX=9606 GN=HNRNPH3 PE=1 SV=2	5	5.5	46.003
HNRNPH1	tr|G8JLB6|G8JLB6_HUMAN Heterogeneous nuclear ribonucleoprotein H OS=Homo sapiens OX=9606 GN=HNRNPH1 PE=1 SV=1	8	5.5	51.067
STIP1	sp|P31948|STIP1_HUMAN Stress-induced-phosphoprotein 1 OS=Homo sapiens OX=9606 GN=STIP1 PE=1 SV=1	1	5.4	7.3744
PRDX1	tr|A0A0A0MRQ5|A0A0A0MRQ5_HUMAN Peroxiredoxin-1 OS=Homo sapiens	2	5.4	6.1741

	OX=9606 GN=PRDX1 PE=1 SV=1			
PYCR1	tr|J3QL24|J3QL24_HUMAN Pyrroline-5-carboxylate reductase 1, mitochondrial (Fragment) OS=Homo sapiens OX=9606 GN=PYCR1 PE=1 SV=1	1	5.3	6.5806
RPL9	tr|H0Y9R4|H0Y9R4_HUMAN 60S ribosomal protein L9 (Fragment) OS=Homo sapiens OX=9606 GN=RPL9 PE=1 SV=2	2	5.3	6.0271
S100A3	sp|P33764|S10A3_HUMAN Protein S100-A3 OS=Homo sapiens OX=9606 GN=S100A3 PE=1 SV=1	3	5.2	5.8091
HSPA1L	tr|Q53FA3|Q53FA3_HUMAN HSPA1L (Fragment) OS=Homo sapiens OX=9606 GN=HSPA1L PE=1 SV=1	0	5.2	6.8705
HSPA4	tr|A0A087WTS8|A0A087WTS8_HUMAN Heat shock 70 kDa protein 4 OS=Homo sapiens OX=9606 GN=HSPA4 PE=1 SV=1	1	5.1	5.8556
PHB	tr|C9JZ20|C9JZ20_HUMAN Prohibitin (Fragment) OS=Homo sapiens OX=9606 GN=PHB PE=1 SV=1	2	5.1	5.6771
KRT9	sp|P35527|K1C9_HUMAN Keratin, type I cytoskeletal 9 OS=Homo sapiens OX=9606 GN=KRT9 PE=1 SV=3	56	5	295.69
MYH9	sp|P35579|MYH9_HUMAN Myosin-9 OS=Homo sapiens OX=9606 GN=MYH9 PE=1 SV=4	21	5	215.62
MYH10	sp|P35580|MYH10_HUMAN Myosin-10 OS=Homo sapiens OX=9606 GN=MYH10 PE=1 SV=3	2	5	11.835
FUS	sp|P35637|FUS_HUMAN RNA-binding protein FUS OS=Homo sapiens OX=9606 GN=FUS PE=1 SV=1	3	4.9	27.709
KRT2	sp|P35908|K22E_HUMAN Keratin, type II cytoskeletal 2 epidermal OS=Homo sapiens OX=9606 GN=KRT2 PE=1 SV=2	58	4.9	323.31
ATP5F1C	sp|P36542|ATPG_HUMAN ATP synthase subunit gamma, mitochondrial OS=Homo sapiens OX=9606 GN=ATP5F1C PE=1 SV=1	1	4.9	5.6216
RPL4	sp|P36578|RL4_HUMAN 60S ribosomal protein L4 OS=Homo sapiens OX=9606 GN=RPL4 PE=1 SV=5	10	4.9	33.14
HSPA9	sp|P38646|GRP75_HUMAN Stress-70 protein, mitochondrial OS=Homo sapiens	1	4.7	6.5224

	OX=9606 GN=HSPA9 PE=1 SV=2			
RPS19	sp|P39019|RS19_HUMAN 40S ribosomal protein S19 OS=Homo sapiens OX=9606 GN=RPS19 PE=1 SV=2	5	4.7	39.216
RBMX	tr|H3BNC1|H3BNC1_HUMAN RNA-binding motif protein, X chromosome OS=Homo sapiens OX=9606 GN=RBMX PE=1 SV=1	3	4.7	65.325
RPL3	tr|B5MCW2|B5MCW2_HUMAN 60S ribosomal protein L3 (Fragment) OS=Homo sapiens OX=9606 GN=RPL3 PE=1 SV=1	2	4.7	5.8688
FEN1	tr|F5H1Y3|F5H1Y3_HUMAN Flap endonuclease 1 (Fragment) OS=Homo sapiens OX=9606 GN=FEN1 PE=1 SV=1	1	4.7	5.6513
CCT6A	sp|P40227|TCPZ_HUMAN T-complex protein 1 subunit zeta OS=Homo sapiens OX=9606 GN=CCT6A PE=1 SV=3	3	4.7	28.065
RPL13A	tr|M0QZU1|M0QZU1_HUMAN 60S ribosomal protein L13a OS=Homo sapiens OX=9606 GN=RPL13A PE=1 SV=1	2	4.6	8.3381
MDH2	sp|P40926|MDHM_HUMAN Malate dehydrogenase, mitochondrial OS=Homo sapiens OX=9606 GN=MDH2 PE=1 SV=3	4	4.4	13.454
TMPO	tr|G5E972|G5E972_HUMAN Lamina-associated polypeptide 2, isoforms beta/gamma OS=Homo sapiens OX=9606 GN=TMPO PE=1 SV=1	1	4.3	5.8387
EIF2S3	tr|H7BZU1|H7BZU1_HUMAN Eukaryotic translation initiation factor 2 subunit 3 (Fragment) OS=Homo sapiens OX=9606 GN=EIF2S3 PE=1 SV=1	4	4.3	16.152
RPL35	tr|F2Z388|F2Z388_HUMAN 60S ribosomal protein L35 OS=Homo sapiens OX=9606 GN=RPL35 PE=1 SV=1	2	4.3	7.5124
RPL27A	tr|E9PJD9|E9PJD9_HUMAN 60S ribosomal protein L27a OS=Homo sapiens OX=9606 GN=RPL27A PE=1 SV=1	2	4.3	5.6743
RPS10-NUDT3	tr|A0A2R8Y6L3|A0A2R8Y6L3_HUMAN RPS10-NUDT3 readthrough (Fragment) OS=Homo sapiens OX=9606 GN=RPS10-NUDT3 PE=4 SV=1	1	4.2	5.7753
CCT5	tr|E7ENZ3|E7ENZ3_HUMAN T-complex protein 1 subunit epsilon OS=Homo sapiens OX=9606 GN=CCT5 PE=1 SV=1	1	4.1	10.809

FASN	tr|A0A0U1RQF0|A0A0U1RQF0_HUMAN Fatty acid synthase OS=Homo sapiens OX=9606 GN=FASN PE=1 SV=1	3	3.9	20.356
CCT3	tr|B4DUR8|B4DUR8_HUMAN T-complex protein 1 subunit gamma OS=Homo sapiens OX=9606 GN=CCT3 PE=1 SV=1	7	3.9	47.095
TUFM	sp|P49411|EFTU_HUMAN Elongation factor Tu, mitochondrial OS=Homo sapiens OX=9606 GN=TUFM PE=1 SV=2	2	3.9	31.369
LIG3	tr|K7EQB6|K7EQB6_HUMAN DNA ligase 3 (Fragment) OS=Homo sapiens OX=9606 GN=LIG3 PE=1 SV=1	1	3.7	5.6493
EMD	sp|P50402|EMD_HUMAN Emerin OS=Homo sapiens OX=9606 GN=EMD PE=1 SV=1	3	3.7	13.922
SERPINH1	tr|E9PLA6|E9PLA6_HUMAN Serpin H1 (Fragment) OS=Homo sapiens OX=9606 GN=SERPINH1 PE=1 SV=8	2	3.7	28.93
CCT8	sp|P50990|TCPQ_HUMAN T-complex protein 1 subunit theta OS=Homo sapiens OX=9606 GN=CCT8 PE=1 SV=4	2	3.6	6.5109
RPL14	tr|E7EPB3|E7EPB3_HUMAN 60S ribosomal protein L14 OS=Homo sapiens OX=9606 GN=RPL14 PE=1 SV=1	4	3.6	28.748
CCT4	sp|P50991|TCPD_HUMAN T-complex protein 1 subunit delta OS=Homo sapiens OX=9606 GN=CCT4 PE=1 SV=4	3	3.5	12.654
HNRNPA3	sp|P51991|ROA3_HUMAN Heterogeneous nuclear ribonucleoprotein A3 OS=Homo sapiens OX=9606 GN=HNRNPA3 PE=1 SV=2	2	3.3	12.866
HNRNPM	tr|M0R019|M0R019_HUMAN Heterogeneous nuclear ribonucleoprotein M (Fragment) OS=Homo sapiens OX=9606 GN=HNRNPM PE=1 SV=8	2	3.3	29.234
SUB1	sp|P53999|TCP4_HUMAN Activated RNA polymerase II transcriptional coactivator p15 OS=Homo sapiens OX=9606 GN=SUB1 PE=1 SV=3	1	3.3	5.962
ALDH18A1	sp|P54886|P5CS_HUMAN Delta-1-pyrroline-5-carboxylate synthase OS=Homo sapiens OX=9606 GN=ALDH18A1 PE=1 SV=2	3	3.3	5.9608
PER3	tr|Q8TAR6|Q8TAR6_HUMAN PER3	3	3.3	5.5936

	protein OS=Homo sapiens OX=9606 GN=PER3 PE=1 SV=1			
TPI1	sp|P60174|TPIS_HUMAN Triosephosphate isomerase OS=Homo sapiens OX=9606 GN=TPI1 PE=1 SV=3	4	3.2	15.799
MYL6	tr|F8W1R7|F8W1R7_HUMAN Myosin light polypeptide 6 OS=Homo sapiens OX=9606 GN=MYL6 PE=1 SV=1	3	3	18.822
ACTG1	sp|P63261|ACTG_HUMAN Actin, cytoplasmic 2 OS=Homo sapiens OX=9606 GN=ACTG1 PE=1 SV=1	22	2.8	175.73
EIF4A1	tr|J3QR64|J3QR64_HUMAN Eukaryotic initiation factor 4A-I (Fragment) OS=Homo sapiens OX=9606 GN=EIF4A1 PE=1 SV=1	4	2.8	13.235
RPS20	tr|E5RIP1|E5RIP1_HUMAN 40S ribosomal protein S20 OS=Homo sapiens OX=9606 GN=RPS20 PE=1 SV=1	1	2.8	5.7967
RPS3A	tr|D6RB09|D6RB09_HUMAN 40S ribosomal protein S3a (Fragment) OS=Homo sapiens OX=9606 GN=RPS3A PE=1 SV=8	3	2.7	19.755
RPL15	tr|E7EX53|E7EX53_HUMAN Ribosomal protein L15 (Fragment) OS=Homo sapiens OX=9606 GN=RPL15 PE=1 SV=1	2	2.6	5.985
RPL26	tr|J3QRI7|J3QRI7_HUMAN 60S ribosomal protein L26 (Fragment) OS=Homo sapiens OX=9606 GN=RPL26 PE=4 SV=1	2	2.6	6.6187
HSPE1	tr|B8ZZL8|B8ZZL8_HUMAN 10 kDa heat shock protein, mitochondrial OS=Homo sapiens OX=9606 GN=HSPE1 PE=1 SV=1	3	2.6	5.6914
HNRNPK	sp|P61978|HNRPK_HUMAN Heterogeneous nuclear ribonucleoprotein K OS=Homo sapiens OX=9606 GN=HNRNPK PE=1 SV=1	17	2.5	118.22
RPS8	tr|Q5JR95|Q5JR95_HUMAN 40S ribosomal protein S8 OS=Homo sapiens OX=9606 GN=RPS8 PE=1 SV=1	6	2.4	30.086
RPS14	tr|E5RH77|E5RH77_HUMAN 40S ribosomal protein S14 OS=Homo sapiens OX=9606 GN=RPS14 PE=1 SV=1	1	2.3	8.8877
RPS16	tr|M0R3H0|M0R3H0_HUMAN 40S ribosomal protein S16 OS=Homo sapiens OX=9606 GN=RPS16 PE=1 SV=1	1	2.3	5.5884
RPS23	sp|P62266|RS23_HUMAN 40S ribosomal protein S23 OS=Homo sapiens OX=9606	1	2.2	5.6637

	GN=RPS23 PE=1 SV=3			
RPS18	sp|P62269|RS18_HUMAN 40S ribosomal protein S18 OS=Homo sapiens OX=9606 GN=RPS18 PE=1 SV=3	3	2.2	23.757
RPS13	sp|P62277|RS13_HUMAN 40S ribosomal protein S13 OS=Homo sapiens OX=9606 GN=RPS13 PE=1 SV=2	5	2.1	25.48
RPS11	tr|M0QZC5|M0QZC5_HUMAN 40S ribosomal protein S11 OS=Homo sapiens OX=9606 GN=RPS11 PE=1 SV=1	2	2.1	5.5886
RPS6	tr|A2A3R5|A2A3R5_HUMAN 40S ribosomal protein S6 OS=Homo sapiens OX=9606 GN=RPS6 PE=1 SV=1	2	2	6.988
RPS23A	tr|K7EMA7|K7EMA7_HUMAN 60S ribosomal protein L23a OS=Homo sapiens OX=9606 GN=RPL23A PE=1 SV=1	3	2	6.6652
H4C1	sp|P62805|H4_HUMAN Histone H4 OS=Homo sapiens OX=9606 GN=H4C1 PE=1 SV=2	6	1.9	14.456
RAN	tr|H0YFC6|H0YFC6_HUMAN GTP-binding nuclear protein Ran (Fragment) OS=Homo sapiens OX=9606 GN=RAN PE=1 SV=1	4	1.9	12.225
RPS24	tr|A0A2R8Y8A0|A0A2R8Y8A0_HUMAN 40S ribosomal protein S24 (Fragment) OS=Homo sapiens OX=9606 GN=RPS24 PE=1 SV=1	1	1.9	5.8097
RPS25	sp|P62851|RS25_HUMAN 40S ribosomal protein S25 OS=Homo sapiens OX=9606 GN=RPS25 PE=1 SV=1	3	1.7	5.7194
FAU	sp|P62861|RS30_HUMAN 40S ribosomal protein S30 OS=Homo sapiens OX=9606 GN=FAU PE=1 SV=1	1	1.7	5.6914
RPL31	tr|B8ZZK4|B8ZZK4_HUMAN 60S ribosomal protein L31 OS=Homo sapiens OX=9606 GN=RPL31 PE=1 SV=1	1	1.7	6.5131
RPL10A	sp|P62906|RL10A_HUMAN 60S ribosomal protein L10a OS=Homo sapiens OX=9606 GN=RPL10A PE=1 SV=2	1	1.7	5.8701
RPL11	tr|Q5VVC8|Q5VVC8_HUMAN 60S ribosomal protein L11 OS=Homo sapiens OX=9606 GN=RPL11 PE=1 SV=2	1	1.6	7.9028
RPL8	tr|E9PP36|E9PP36_HUMAN 60S ribosomal protein L8 OS=Homo sapiens OX=9606 GN=RPL8 PE=1 SV=1	3	1.5	5.6314

PPIA	tr|E5RIZ5|E5RIZ5_HUMAN Peptidyl-prolyl cis-trans isomerase A OS=Homo sapiens OX=9606 GN=PPIA PE=1 SV=1	2	1.3	17.614
YWHAZ	sp|P63104|1433Z_HUMAN 14-3-3 protein zeta/delta OS=Homo sapiens OX=9606 GN=YWHAZ PE=1 SV=1	2	1.2	37.083
EIF5A	tr|I3L397|I3L397_HUMAN Eukaryotic translation initiation factor 5A (Fragment) OS=Homo sapiens OX=9606 GN=EIF5A PE=1 SV=8	2	1	11.888
RACK1	sp|P63244|RACK1_HUMAN Receptor of activated protein C kinase 1 OS=Homo sapiens OX=9606 GN=RACK1 PE=1 SV=3	4	0.9	24.91
EEF1A1P5	sp|Q5VTE0|EF1A3_HUMAN Putative elongation factor 1-alpha-like 3 OS=Homo sapiens OX=9606 GN=EEF1A1P5 PE=5 SV=1	15	0.8	77.173
TUBA1B	sp|P68363|TBA1B_HUMAN Tubulin alpha-1B chain OS=Homo sapiens OX=9606 GN=TUBA1B PE=1 SV=1	2	0.8	104.34
TUBB4B	sp|P68371|TBB4B_HUMAN Tubulin beta-4B chain OS=Homo sapiens OX=9606 GN=TUBB4B PE=1 SV=1	6	0.7	61.381
H3-3B	tr|K7EMV3|K7EMV3_HUMAN Histone H3 OS=Homo sapiens OX=9606 GN=H3-3B PE=1 SV=1	1	0.7	5.9322
CCT2	tr|F8VQ14|F8VQ14_HUMAN T-complex protein 1 subunit beta OS=Homo sapiens OX=9606 GN=CCT2 PE=1 SV=1	1	0.5	6.3568
DCD	sp|P81605|DCD_HUMAN Dermcidin OS=Homo sapiens OX=9606 GN=DCD PE=1 SV=2	5	0.5	12.808
CLTC	sp|Q00610|CLH1_HUMAN Clathrin heavy chain 1 OS=Homo sapiens OX=9606 GN=CLTC PE=1 SV=5	3	0.4	6.5131
RPL19	tr|J3QR09|J3QR09_HUMAN Ribosomal protein L19 OS=Homo sapiens OX=9606 GN=RPL19 PE=1 SV=1	3	0.4	18.859
HNRNPU	tr|Q5RI18|Q5RI18_HUMAN Heterogeneous nuclear ribonucleoprotein U OS=Homo sapiens OX=9606 GN=HNRNPU PE=1 SV=9	4	0	11.473
SPTBN1	sp|Q01082|SPTB2_HUMAN Spectrin beta chain, non-erythrocytic 1 OS=Homo sapiens	3	0	6.0835

	OX=9606 GN=SPTBN1 PE=1 SV=2			
EWSR1	tr|A0A0D9SFL3|A0A0D9SFL3_HUMAN RNA-binding protein EWS OS=Homo sapiens OX=9606 GN=EWSR1 PE=1 SV=1	5	0	44.033
RPL6	sp|Q02878|RL6_HUMAN 60S ribosomal protein L6 OS=Homo sapiens OX=9606 GN=RPL6 PE=1 SV=3	3	0	5.7226
SSBP1	sp|Q04837|SSBP_HUMAN Single-stranded DNA-binding protein, mitochondrial OS=Homo sapiens OX=9606 GN=SSBP1 PE=1 SV=1	2	0	11.758
AHNAK	sp|Q09666|AHNK_HUMAN Neuroblast differentiation-associated protein AHNAK OS=Homo sapiens OX=9606 GN=AHNAK PE=1 SV=2	2	0	8.2182
DNAH9	tr|E7EP17|E7EP17_HUMAN Dynein heavy chain 9, axonemal OS=Homo sapiens OX=9606 GN=DNAH9 PE=1 SV=1	14	0	48.535
HRNR	sp|Q86YZ3|HORN_HUMAN Hornerin OS=Homo sapiens OX=9606 GN=HRNR PE=1 SV=2	4	0	36.788
GOLGA4	tr|H0Y6I0|H0Y6I0_HUMAN Golgin subfamily A member 4 (Fragment) OS=Homo sapiens OX=9606 GN=GOLGA4 PE=1 SV=1	1	0	5.6123
MYH14	sp|Q7Z406|MYH14_HUMAN Myosin-14 OS=Homo sapiens OX=9606 GN=MYH14 PE=1 SV=2	1	0	5.5842
ARHGEF17	sp|Q96PE2|ARHGH_HUMAN Rho guanine nucleotide exchange factor 17 OS=Homo sapiens OX=9606 GN=ARHGEF17 PE=1 SV=1	1	0	5.6402
NUP210	sp|Q8TEM1|PO210_HUMAN Nuclear pore membrane glycoprotein 210 OS=Homo sapiens OX=9606 GN=NUP210 PE=1 SV=3	2	0	11.683
KIF26A	tr|C9JFF0|C9JFF0_HUMAN Kinesin-like protein KIF26A OS=Homo sapiens OX=9606 GN=KIF26A PE=1 SV=1	1	0	5.6979
ARHGEF12	tr|E9PMR6|E9PMR6_HUMAN Rho guanine nucleotide exchange factor 12 OS=Homo sapiens OX=9606 GN=ARHGEF12 PE=1 SV=1	1	0	5.8375
KANK1	sp|Q14678|KANK1_HUMAN KN motif and ankyrin repeat domain-containing protein 1	4	0	17.723

	OS=Homo sapiens OX=9606 GN=KANK1 PE=1 SV=3			
DHX9	sp|Q08211|DHX9_HUMAN ATP-dependent RNA helicase A OS=Homo sapiens OX=9606 GN=DHX9 PE=1 SV=4	1	0	5.7858
ABCB5	sp|Q2M3G0|ABCB5_HUMAN ATP-binding cassette sub-family B member 5 OS=Homo sapiens OX=9606 GN=ABCB5 PE=1 SV=4	4	0	25.456
WDR11	sp|Q9BZH6|WDR11_HUMAN WD repeat-containing protein 11 OS=Homo sapiens OX=9606 GN=WDR11 PE=1 SV=1	1	0	5.8048
MN1	sp|Q10571|MN1_HUMAN Transcriptional activator MN1 OS=Homo sapiens OX=9606 GN=MN1 PE=1 SV=3	2	0	18.198
SORCS1	sp|Q8WY21|SORC1_HUMAN VPS10 domain-containing receptor SorCS1 OS=Homo sapiens OX=9606 GN=SORCS1 PE=1 SV=3	3	0	16.078
AP4E1	sp|Q9UPM8|AP4E1_HUMAN AP-4 complex subunit epsilon-1 OS=Homo sapiens OX=9606 GN=AP4E1 PE=1 SV=2	3	0	12.624
FRMD4A	sp|Q9P2Q2|FRM4A_HUMAN FERM domain-containing protein 4A OS=Homo sapiens OX=9606 GN=FRMD4A PE=1 SV=3	2	0	13.295
UBAP2L	sp|Q14157|UBP2L_HUMAN Ubiquitin-associated protein 2-like OS=Homo sapiens OX=9606 GN=UBAP2L PE=1 SV=2	3	0	6.2838
FNBP4	sp|Q8N3X1|FNBP4_HUMAN Formin-binding protein 4 OS=Homo sapiens OX=9606 GN=FNBP4 PE=1 SV=3	5	0	19.879
XRN2	sp|Q9H0D6|XRN2_HUMAN 5-3 exoribonuclease 2 OS=Homo sapiens OX=9606 GN=XRN2 PE=1 SV=1	4	0	17.915
CUL4B	sp|Q13620|CUL4B_HUMAN Cullin-4B OS=Homo sapiens OX=9606 GN=CUL4B PE=1 SV=4	1	0	6.0044
ZZZ3	sp|Q8IYH5|ZZZ3_HUMAN ZZ-type zinc finger-containing protein 3 OS=Homo sapiens OX=9606 GN=ZZZ3 PE=1 SV=1	1	0	5.6821
TBCK	sp|Q8TEA7|TBCK_HUMAN TBC domain-containing protein kinase-like protein OS=Homo sapiens OX=9606 GN=TBCK PE=1 SV=4	3	0	6.8087

PSMD2	sp|Q13200|PSMD2_HUMAN 26S proteasome non-ATPase regulatory subunit 2 OS=Homo sapiens OX=9606 GN=PSMD2 PE=1 SV=3	1	0	6.654
ZN598	sp|Q86UK7|ZN598_HUMAN E3 ubiquitin-protein ligase ZNF598 OS=Homo sapiens OX=9606 GN=ZNF598 PE=1 SV=1	1	0	5.9775
HELB	tr|F5H1I4|F5H1I4_HUMAN DNA helicase B OS=Homo sapiens OX=9606 GN=HELB PE=1 SV=1	1	0	5.9129
FSTL4	sp|Q6MZW2|FSTL4_HUMAN Follistatin-related protein 4 OS=Homo sapiens OX=9606 GN=FSTL4 PE=2 SV=3	3	0	5.5988
FAM160A2	tr|E9PJK5|E9PJK5_HUMAN FTS and Hook-interacting protein OS=Homo sapiens OX=9606 GN=FAM160A2 PE=1 SV=1	3	0	6.2352
WDR7	tr|A2RRE0|A2RRE0_HUMAN WD repeat-containing protein 7 OS=Homo sapiens OX=9606 GN=WDR7 PE=1 SV=1	3	0	6.2226
ARHGEF38	sp|Q9NXL2|ARH38_HUMAN Rho guanine nucleotide exchange factor 38 OS=Homo sapiens OX=9606 GN=ARHGEF38 PE=2 SV=2	1	0	7.2994
IRF2BPL	sp|Q9H1B7|I2BPL_HUMAN Probable E3 ubiquitin-protein ligase IRF2BPL OS=Homo sapiens OX=9606 GN=IRF2BPL PE=1 SV=1	1	0	7.1534
GALNT7	sp|Q86SF2|GALT7_HUMAN N-acetylgalactosaminyltransferase 7 OS=Homo sapiens OX=9606 GN=GALNT7 PE=1 SV=1	1	0	5.6157
DDX1	tr|F1T0B3|F1T0B3_HUMAN ATP-dependent RNA helicase DDX1 OS=Homo sapiens OX=9606 GN=DDX1 PE=1 SV=1	2	0	5.72
DNAH10	tr|F5H515|F5H515_HUMAN Dynein heavy chain 10, axonemal (Fragment) OS=Homo sapiens OX=9606 GN=DNAH10 PE=1 SV=2	1	0	5.707
ATAD3A	sp|Q9NVI7|ATD3A_HUMAN ATPase family AAA domain-containing protein 3A OS=Homo sapiens OX=9606 GN=ATAD3A PE=1 SV=2	1	0	5.5871
PABPC4	sp|Q13310|PABP4_HUMAN Polyadenylate-binding protein 4 OS=Homo sapiens	1	0	7.0446

	OX=9606 GN=PABPC4 PE=1 SV=1			
COL18A1	tr|H7BXV5|H7BXV5_HUMAN Collagen alpha-1(XVIII) chain (Fragment) OS=Homo sapiens OX=9606 GN=COL18A1 PE=1 SV=1	1	0	6.3196
RBM14	sp|Q96PK6|RBM14_HUMAN RNA-binding protein 14 OS=Homo sapiens OX=9606 GN=RBM14 PE=1 SV=2	1	0	6.004
IMMT	tr|H7C463|H7C463_HUMAN MICOS complex subunit MIC60 (Fragment) OS=Homo sapiens OX=9606 GN=IMMT PE=1 SV=1	7	0	47.238
ZC3H12A	sp|Q5D1E8|ZC12A_HUMAN Endoribonuclease ZC3H12A OS=Homo sapiens OX=9606 GN=ZC3H12A PE=1 SV=1	1	0	5.8951
PGPS1	sp|Q32NB8|PGPS1_HUMAN CDP-diacylglycerol--glycerol-3-phosphate 3-phosphatidyltransferase, mitochondrial OS=Homo sapiens OX=9606 GN=PGS1 PE=2 SV=1	1	0	28.461
TMEM62	tr|H3BVF7|H3BVF7_HUMAN Transmembrane protein 62 (Fragment) OS=Homo sapiens OX=9606 GN=TMEM62 PE=1 SV=1	2	0	7.8621
SVOP	sp|Q8N4V2|SVOP_HUMAN Synaptic vesicle 2-related protein OS=Homo sapiens OX=9606 GN=SVOP PE=2 SV=1	1	0	5.8095
CCT7	sp|Q99832|TCPH_HUMAN T-complex protein 1 subunit eta OS=Homo sapiens OX=9606 GN=CCT7 PE=1 SV=2	3	0	5.7839
CHD3	tr|H7C2H0|H7C2H0_HUMAN Chromodomain-helicase-DNA-binding protein 3 (Fragment) OS=Homo sapiens OX=9606 GN=CHD3 PE=1 SV=1	2	0	7.4032
PSPC1	sp|Q8WXF1|PSPC1_HUMAN Paraspeckle component 1 OS=Homo sapiens OX=9606 GN=PSPC1 PE=1 SV=1	3	0	5.6625
SPATA2	sp|Q9UM82|SPAT2_HUMAN Spermatogenesis-associated protein 2 OS=Homo sapiens OX=9606 GN=SPATA2 PE=1 SV=2	1	0	5.9432
API5	tr|G3V1C3|G3V1C3_HUMAN Apoptosis inhibitor 5 OS=Homo sapiens OX=9606	1	0	5.6157

	GN=API5 PE=1 SV=1			
SOWAHA	sp|Q2M3V2|SWAHA_HUMAN Ankyrin repeat domain-containing protein SOWAHA OS=Homo sapiens OX=9606 GN=SOWAHA PE=1 SV=3	1	0	5.9149
CCDC65	sp|Q8IXS2|DRC2_HUMAN Dynein regulatory complex subunit 2 OS=Homo sapiens OX=9606 GN=CCDC65 PE=1 SV=2	1	0	5.5986
SRRT	tr|H7C3A1|H7C3A1_HUMAN Serrate RNA effector molecule homolog (Fragment) OS=Homo sapiens OX=9606 GN=SRRT PE=1 SV=1	1	0	5.5966
SLC30A1	sp|Q9Y6M5|ZNT1_HUMAN Zinc transporter 1 OS=Homo sapiens OX=9606 GN=SLC30A1 PE=1 SV=3	3	0	13.08
RTCB	sp|Q9Y3I0|RTCB_HUMAN RNA-splicing ligase RtcB homolog OS=Homo sapiens OX=9606 GN=RTCB PE=1 SV=1	2	0	15.462
NONO	sp|Q15233|NONO_HUMAN Non-POU domain-containing octamer-binding protein OS=Homo sapiens OX=9606 GN=NONO PE=1 SV=4	1	0	5.585
G3BP2	sp|Q9UN86|G3BP2_HUMAN Ras GTPase-activating protein-binding protein 2 OS=Homo sapiens OX=9606 GN=G3BP2 PE=1 SV=2	3	0	5.7499
CALCRL	sp|Q16602|CALRL_HUMAN Calcitonin gene-related peptide type 1 receptor OS=Homo sapiens OX=9606 GN=CALCRL PE=1 SV=2	3	0	6.3196
ANKRD34A	sp|Q69YU3|AN34A_HUMAN Ankyrin repeat domain-containing protein 34A OS=Homo sapiens OX=9606 GN=ANKRD34A PE=1 SV=2	1	0	6.71
CPSF6	tr|F8WJN3|F8WJN3_HUMAN Cleavage and polyadenylation-specificity factor subunit 6 OS=Homo sapiens OX=9606 GN=CPSF6 PE=1 SV=1	1	0	6.4488
G3BP1	sp|Q13283|G3BP1_HUMAN Ras GTPase-activating protein-binding protein 1 OS=Homo sapiens OX=9606 GN=G3BP1 PE=1 SV=1	3	0	21.042
FIZ1	sp|Q96SL8|FIZ1_HUMAN Flt3-interacting	1	0	5.7331

	zinc finger protein 1 OS=Homo sapiens OX=9606 GN=FIZ1 PE=1 SV=2			
TUBA1C	sp|Q9BQE3|TBA1C_HUMAN Tubulin alpha-1C chain OS=Homo sapiens OX=9606 GN=TUBA1C PE=1 SV=1	1	0	7.8621
SDE2	sp|Q6IQ49|SDE2_HUMAN Replication stress response regulator SDE2 OS=Homo sapiens OX=9606 GN=SDE2 PE=1 SV=1	3	0	22.831
TBC1D10C	sp|Q8IV04|TB10C_HUMAN Carabin OS=Homo sapiens OX=9606 GN=TBC1D10C PE=1 SV=1	2	0	5.9351
APOL2	tr|J3KQL8|J3KQL8_HUMAN Apolipoprotein L2 OS=Homo sapiens OX=9606 GN=APOL2 PE=1 SV=2	2	0	7.3228
TAF15	tr|A0A075B7D9|A0A075B7D9_HUMAN TATA-binding protein-associated factor 2N OS=Homo sapiens OX=9606 GN=TAF15 PE=1 SV=1	3	0	5.6603
KHDRBS1	sp|Q07666|KHDR1_HUMAN KH domain-containing, RNA-binding, signal transduction-associated protein 1 OS=Homo sapiens OX=9606 GN=KHDRBS1 PE=1 SV=1	13	0	62.86
TOR4A	sp|Q9NXH8|TOR4A_HUMAN Torsin-4A OS=Homo sapiens OX=9606 GN=TOR4A PE=1 SV=2	2	0	5.5842
SERBP1	sp|Q8NC51|PAIRB_HUMAN Plasminogen activator inhibitor 1 RNA-binding protein OS=Homo sapiens OX=9606 GN=SERBP1 PE=1 SV=2	2	0	14.302
RXFP4	sp|Q8TDU9|RL3R2_HUMAN Relaxin-3 receptor 2 OS=Homo sapiens OX=9606 GN=RXFP4 PE=1 SV=1	3	0	18.766
SUGT1	sp|Q9Y2Z0|SGT1_HUMAN Protein SGT1 homolog OS=Homo sapiens OX=9606 GN=SUGT1 PE=1 SV=3	2	0	5.7406
ILF2	tr|B4DY09|B4DY09_HUMAN Interleukin enhancer-binding factor 2 OS=Homo sapiens OX=9606 GN=ILF2 PE=1 SV=1	2	0	7.3661
PCBP1	sp|Q15365|PCBP1_HUMAN Poly(rC)-binding protein 1 OS=Homo sapiens OX=9606 GN=PCBP1 PE=1 SV=2	2	0	11.981
GLS2	tr|A0A087X004|A0A087X004_HUMAN Glutaminase 2 (Liver, mitochondrial),	2	0	5.9732

	isoform CRA_d OS=Homo sapiens OX=9606 GN=GLS2 PE=1 SV=1			
AAK1	tr|A0A096LNZ0|A0A096LNZ0_HUMAN AP2-associated protein kinase 1 (Fragment) OS=Homo sapiens OX=9606 GN=AAK1 PE=1 SV=6	2	0	11.592
RUVBL1	tr|E7ETR0|E7ETR0_HUMAN RuvB-like helicase OS=Homo sapiens OX=9606 GN=RUVBL1 PE=1 SV=1	3	0	6.3742
FAM98A	tr|E9PH82|E9PH82_HUMAN Protein FAM98A OS=Homo sapiens OX=9606 GN=FAM98A PE=1 SV=1	6	0	18.614
GIPC2	sp|Q8TF65|GIPC2_HUMAN PDZ domain-containing protein GIPC2 OS=Homo sapiens OX=9606 GN=GIPC2 PE=1 SV=1	1	0	5.9999
TMEM200B	sp|Q69YZ2|T200B_HUMAN Transmembrane protein 200B OS=Homo sapiens OX=9606 GN=TMEM200B PE=2 SV=1	2	0	8.3401
PABPN1	sp|Q86U42|PABP2_HUMAN Polyadenylate-binding protein 2 OS=Homo sapiens OX=9606 GN=PABPN1 PE=1 SV=3	7	0	88.633
HNRNPA0	sp|Q13151|ROA0_HUMAN Heterogeneous nuclear ribonucleoprotein A0 OS=Homo sapiens OX=9606 GN=HNRNPA0 PE=1 SV=1	1	0	5.8962
PRKACB	tr|B1APG3|B1APG3_HUMAN cAMP-dependent protein kinase catalytic subunit beta OS=Homo sapiens OX=9606 GN=PRKACB PE=1 SV=1	1	0	5.8638
HNRNPAB	tr|D6R9P3|D6R9P3_HUMAN Heterogeneous nuclear ribonucleoprotein A/B OS=Homo sapiens OX=9606 GN=HNRNPAB PE=1 SV=1	1	0	5.5946
RTRAF	sp|Q9Y224|RTRAF_HUMAN RNA transcription, translation and transport factor protein OS=Homo sapiens OX=9606 GN=RTRAF PE=1 SV=1	6	0	28.724
FAM122B	sp|Q7Z309|F122B_HUMAN Protein FAM122B OS=Homo sapiens OX=9606 GN=FAM122B PE=1 SV=2	3	0	7.7775
ALYREF	sp|Q86V81|THOC4_HUMAN THO complex subunit 4 OS=Homo sapiens OX=9606 GN=ALYREF PE=1 SV=3	1	0	5.9427

TMEM204	sp|Q9BSN7|TM204_HUMAN Transmembrane protein 204 OS=Homo sapiens OX=9606 GN=TMEM204 PE=2 SV=1	2	0	5.6603
KRT72	tr|H0YIG3|H0YIG3_HUMAN Keratin, type II cytoskeletal 72 (Fragment) OS=Homo sapiens OX=9606 GN=KRT72 PE=4 SV=1	1	0	8.1961
CAPRIN1	tr|E9PLA9|E9PLA9_HUMAN Caprin-1 (Fragment) OS=Homo sapiens OX=9606 GN=CAPRIN1 PE=1 SV=1	2	0	5.6937
SIRT6	tr|M0R1N9|M0R1N9_HUMAN NAD-dependent protein deacetylase sirtuin-6 OS=Homo sapiens OX=9606 GN=SIRT6 PE=1 SV=1	4	0	13.98
MSANTD2	tr|B4E1M0|B4E1M0_HUMAN Myb/SANT-like DNA-binding domain-containing protein 2 OS=Homo sapiens OX=9606 GN=MSANTD2 PE=1 SV=1	1	0	6.3297
PPP1R14B	sp|Q96C90|PP14B_HUMAN Protein phosphatase 1 regulatory subunit 14B OS=Homo sapiens OX=9606 GN=PPP1R14B PE=1 SV=3	1	0	11.004
CEP295	tr|E9PJG3|E9PJG3_HUMAN Centrosomal protein of 295 kDa (Fragment) OS=Homo sapiens OX=9606 GN=CEP295 PE=1 SV=1	5	0	24.942
MRPL51	sp|Q4U2R6|RM51_HUMAN 39S ribosomal protein L51, mitochondrial OS=Homo sapiens OX=9606 GN=MRPL51 PE=1 SV=1	2	0	5.6412
SYPL1	tr|A0A0U1RQT9|A0A0U1RQT9_HUMAN Synaptophysin-like protein 1 (Fragment) OS=Homo sapiens OX=9606 GN=SYPL1 PE=1 SV=1	1	0	7.7399
RPL18	tr|F8VUA6|F8VUA6_HUMAN 60S ribosomal protein L18 (Fragment) OS=Homo sapiens OX=9606 GN=RPL18 PE=1 SV=1	3	0	5.691
GIGYF2	tr|C9IYH5|C9IYH5_HUMAN GRB10-interacting GYF protein 2 (Fragment) OS=Homo sapiens OX=9606 GN=GIGYF2 PE=1 SV=1	1	0	5.6422
SLC24A5	tr|H0YLZ0|H0YLZ0_HUMAN Sodium/potassium/calcium exchanger 5 OS=Homo sapiens OX=9606 GN=SLC24A5	2	0	6.4262

	PE=1 SV=1			
SHMT1	tr|A0A087WTD8|A0A087WTD8_HUMAN Serine hydroxymethyltransferase, cytosolic OS=Homo sapiens OX=9606 GN=SHMT1 PE=1 SV=1	3	0	5.5842
FSCN1	tr|C9JPH9|C9JPH9_HUMAN Fascin (Fragment) OS=Homo sapiens OX=9606 GN=FSCN1 PE=1 SV=1	1	0	5.9938
KANK2	tr|K7ERU2|K7ERU2_HUMAN KN motif and ankyrin repeat domain-containing protein 2 (Fragment) OS=Homo sapiens OX=9606 GN=KANK2 PE=1 SV=1	2	0	15.987
HNRNPD	tr|D6RF44|D6RF44_HUMAN Heterogeneous nuclear ribonucleoprotein D0 (Fragment) OS=Homo sapiens OX=9606 GN=HNRNPD PE=1 SV=8	1	0	5.6531
ALDH16A1	tr|M0QYY1|M0QYY1_HUMAN Aldehyde dehydrogenase family 16 member A1 OS=Homo sapiens OX=9606 GN=ALDH16A1 PE=4 SV=1	2	0	5.7702
PHB2	tr|F5H2D2|F5H2D2_HUMAN Prohibitin OS=Homo sapiens OX=9606 GN=PHB2 PE=1 SV=1	1	0	7.7048
KCNS3	tr|C9J187|C9J187_HUMAN Potassium voltage-gated channel subfamily S member 3 (Fragment) OS=Homo sapiens OX=9606 GN=KCNS3 PE=4 SV=1	2	0	5.5896
COL3A1	tr|H7C435|H7C435_HUMAN Collagen alpha-1(III) chain (Fragment) OS=Homo sapiens OX=9606 GN=COL3A1 PE=1 SV=1	3	0	7.9727
ASAH1	tr|A0A1B0GW30|A0A1B0GW30_HUMAN Acid ceramidase OS=Homo sapiens OX=9606 GN=ASAH1 PE=4 SV=1	2	0	6.0194
AUTS2	tr|H7C2P0|H7C2P0_HUMAN Autism susceptibility gene 2 protein (Fragment) OS=Homo sapiens OX=9606 GN=AUTS2 PE=1 SV=2	1	0	7.0523
TRAJ56	tr|A0A075B6Z2|A0A075B6Z2_HUMAN T cell receptor alpha joining 56 (Fragment) OS=Homo sapiens OX=9606 GN=TRAJ56 PE=4 SV=1	1	0	5.5842

Table S6 Related to Figure 4; TTC7B-related E3 ubiquitin ligase.

Gene-name	Description	Unique peptides	Sequence coverage [%]
NOSIP	tr|M0R3B2|M0R3B2_HUMAN Nitric oxide synthase-interacting protein (Fragment) OS=Homo sapiens OX=9606 GN=NOSIP PE=1 SV=8;sp|Q9Y314|NOSIP_HUMAN Nitric oxide synthase-interacting protein OS=Homo sapiens OX=9606 GN=NOSIP PE=1 SV=1	1	12.4
TRIM21	sp|P19474|RO52_HUMAN E3 ubiquitin-protein ligase TRIM21 OS=Homo sapiens OX=9606 GN=TRIM21 PE=1 SV=1	3	6.1
TRIM4	tr|H7C0Q6|H7C0Q6_HUMAN Tripartite motif containing 4 (Fragment) OS=Homo sapiens OX=9606 GN=TRIM4 PE=1 SV=1;tr|B4DEC5|B4DEC5_HUMAN cDNA FLJ60086, highly similar to Tripartite motif-containing protein 4 OS=Homo sapiens OX=9606 PE=2 SV=1;tr|B3KS62|B3KS62_HUMA	1	5.4
HACE1	tr|E3W983|E3W983_HUMAN HECT domain and ankyrin repeat containing E3 ubiquitin protein ligase 1 OS=Homo sapiens OX=9606 GN=HACE1 PE=1 SV=2;tr|E5RHI1|E5RHI1_HUMAN HECT domain and ankyrin repeat containing E3 ubiquitin protein ligase 1 (Fragment) OS=Homo sapi	1	5.1
WWP2	tr|B4DHF6|B4DHF6_HUMAN HECT-type E3 ubiquitin transferase OS=Homo sapiens OX=9606	1	3.5

	PE=2 SV=1;tr|B4DIN7|B4DIN7_HUMAN E3 ubiquitin-protein ligase OS=Homo sapiens OX=9606 PE=2 SV=1;sp|O00308|WWP2_HUMAN NEDD4-like E3 ubiquitin-protein ligase WWP2 OS=Homo sapien		
UBE2O	sp|Q9C0C9|UBE2O_HUMAN (E3-independent) E2 ubiquitin-conjugating enzyme OS=Homo sapiens OX=9606 GN=UBE2O PE=1 SV=3	1	2.5
ZFP91	sp|Q96JP5|ZFP91_HUMAN E3 ubiquitin-protein ligase ZFP91 OS=Homo sapiens OX=9606 GN=ZFP91 PE=1 SV=1	1	1.6
TRIM34; TRIM6-TRIM34	sp|Q9BYJ4|TRI34_HUMAN E3 ubiquitin-protein ligase TRIM34 OS=Homo sapiens OX=9606 GN=TRIM34 PE=1 SV=2;tr|B2RNG4|B2RNG4_HUMAN TRIM6-TRIM34 readthrough OS=Homo sapiens OX=9606 GN=TRIM6-TRIM34 PE=2 SV=1	1	1.4
HERC2	tr|A0A3B3IRP6|A0A3B3IRP6_HUMAN HECT and RLD domain containing E3 ubiquitin protein ligase 2 (Fragment) OS=Homo sapiens OX=9606 GN=HERC2 PE=1 SV=1;tr|A0A0J9YVP0|A0A0J9YVP0_HUMAN E3 ubiquitin-protein ligase HERC2 (Fragment) OS=Homo sapiens OX=9606 GN=HERC2 P	1	0.9
RNF213	tr|A0A0A0MTR7|A0A0A0MTR7_HUMAN Ring finger protein 213 OS=Homo sapiens OX=9606 GN=RNF213 PE=1 SV=1;sp|Q63HN8|RN213_HUMAN E3 ubiquitin-	3	0.6

protein ligase RNF213 OS=Homo sapiens

OX=9606 GN=RNF213 PE=1

SV=3;tr|A0A0A0MTC1|A0A0A0MTC1_HUMAN

Ring finger protein 213 O

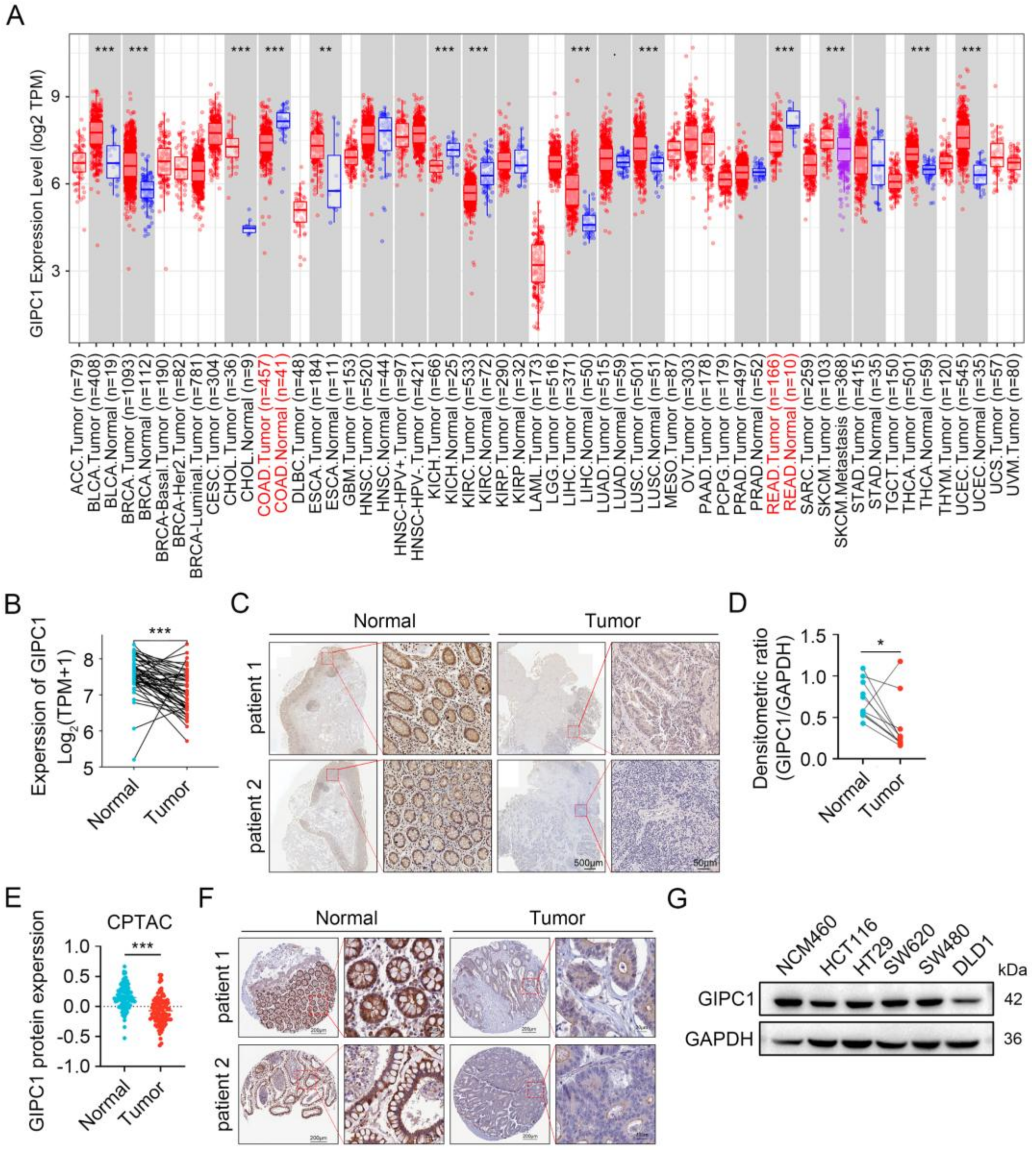

Figure S1. GIPC1 is lowly expressed in colorectal cancer. (A) Analysis of GIPC1 mRNA expression levels in various cancers and corresponding normal tissues using TCGA data from the TIMER website. (B) GIPC1 mRNA expression levels in colorectal cancer tissues and paired normal tissues base on TCGA data. (C) Immunohistochemistry (IHC) staining of collected CRC and paired adjacent normal tissues. Scale bars are shown in Figure S1C. (D) Grayscale quantification of Figure 1F. (E) GIPC1 protein expression levels in colon cancer and adjacent normal tissues on CPTAC website. (F) IHC staining of colon cancer and adjacent normal tissues on HPA

website. Scale bars are shown in Figure S1F. (G) Expression levels of GIPC1 in CRC cells. NCM460: normal colonic epithelial cells. Data are presented as mean ± SD. *P < 0.05, **P < 0.01, ***P < 0.001.

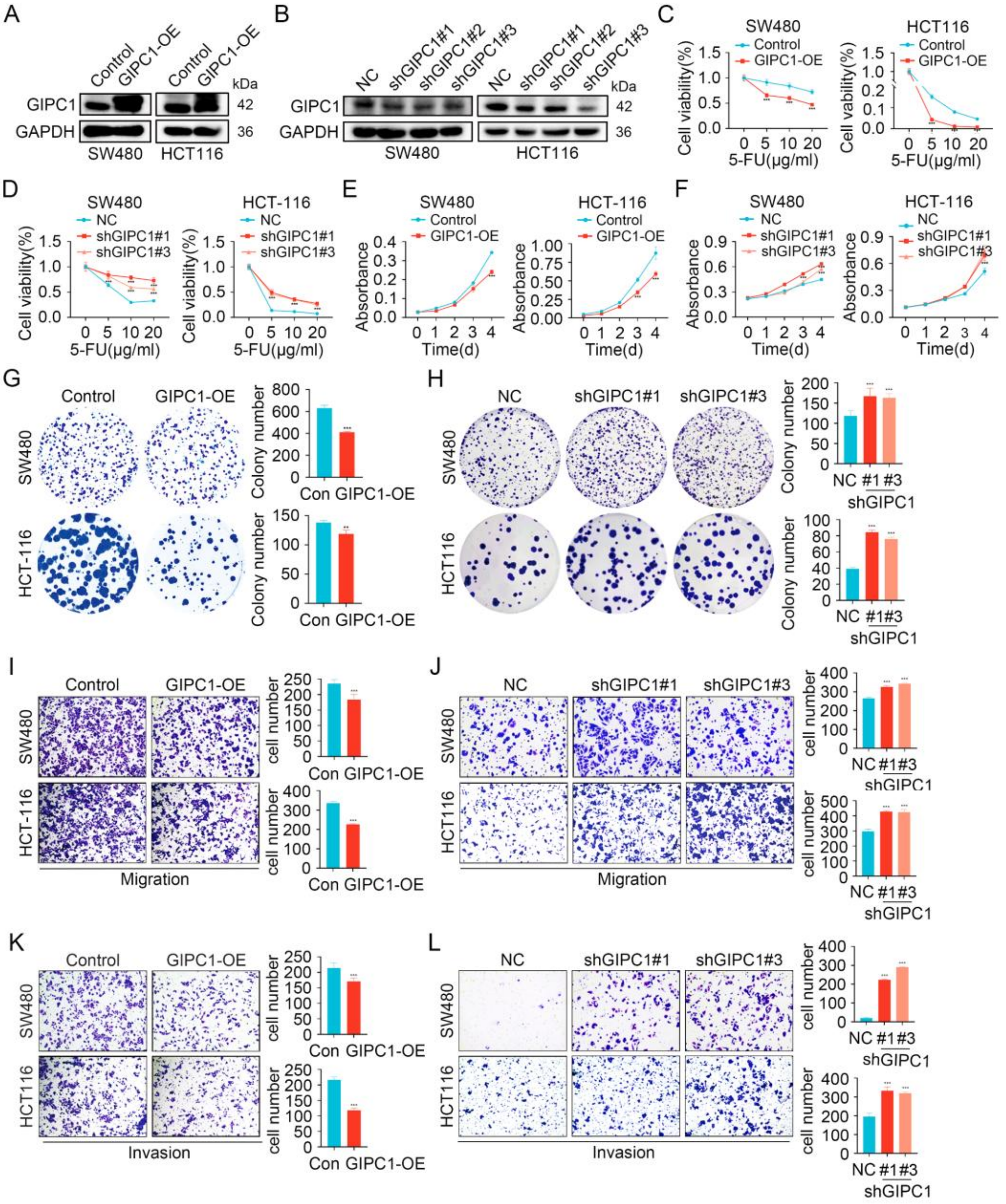

Figure S2. GIPC1 inhibits chemoresistance, proliferation, migration, and invasion. (A) Overexpression of GIPC1 in SW480 and HCT116 cells. (B) Knock down GIPC1 using three independent shRNAs. (C-D) Survival rate of CRC cells measured following exposure to varying concentrations of 5-FU. (E-H) CCK8 and colony formation assays showing the proliferation. (I-L) Transwell assays evaluating cell migratory and invasive capabilities. Data are presented as mean ± SD. **P < 0.01, ***P < 0.001.

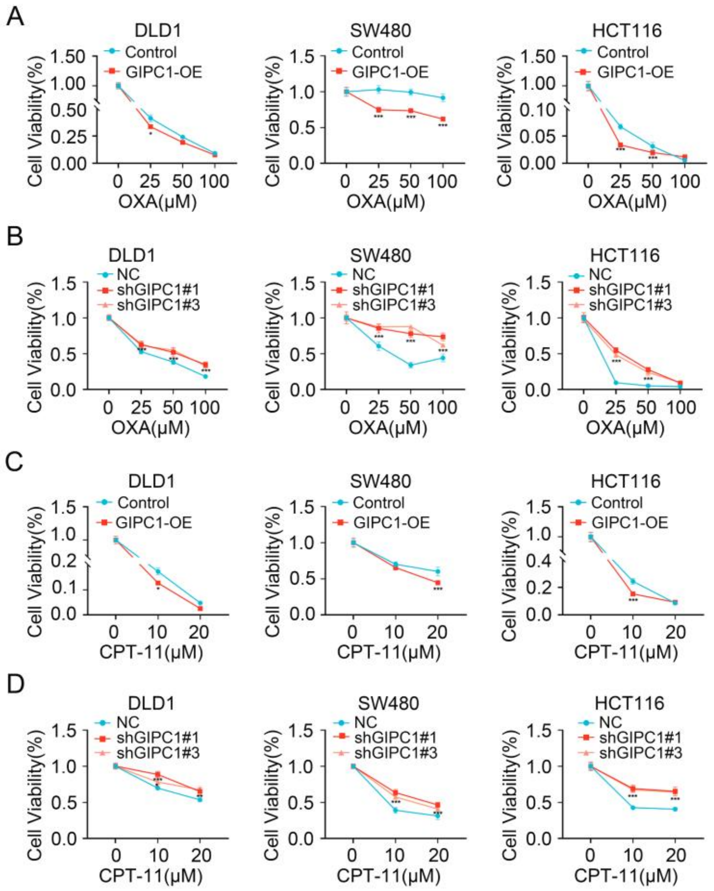

Figure S3. GIPC1 inhibits chemoresistance in colorectal cancer cells. (A-D) Cell viability measured following exposure to varying concentrations of OXA (A-B) or CPT-11 (C-D). Data are presented as mean ± SD. $^{*}P < 0.05$, $^{**}P < 0.01$, $^{***}P < 0.001$.

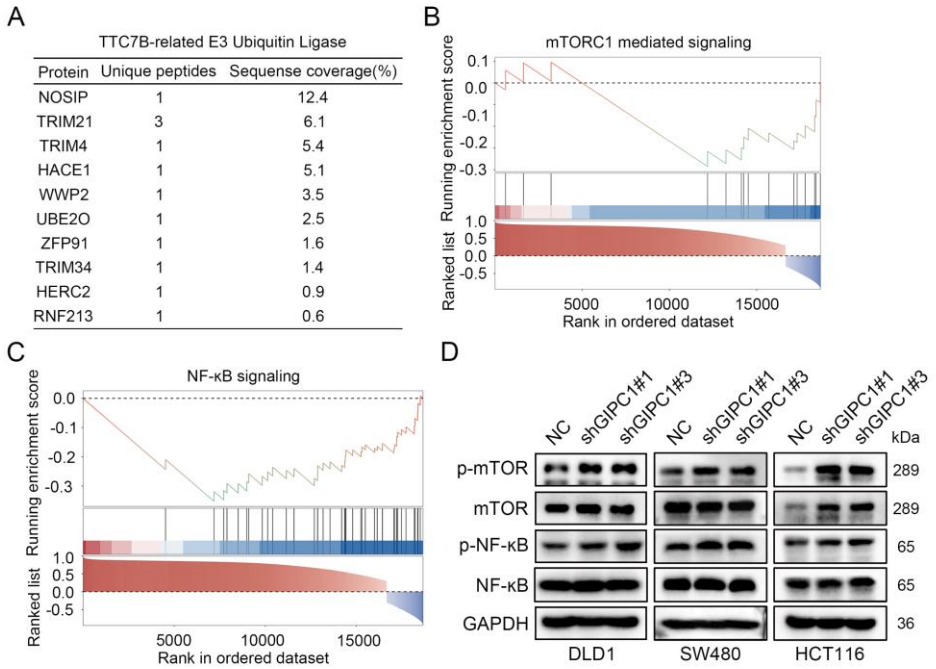

Figure S4. GIPC1 inhibits the mTOR/NF-κB pathway. (A) TTC7B-related E3 ubiquitin ligase. (B-C) GSEA analysis of colorectal cancer data from GSE32323. (D) Detection of mTOR, NF-κB, and their phosphorylation levels in DLD1, SW480, and HCT116 cells with GIPC1 knockdown.

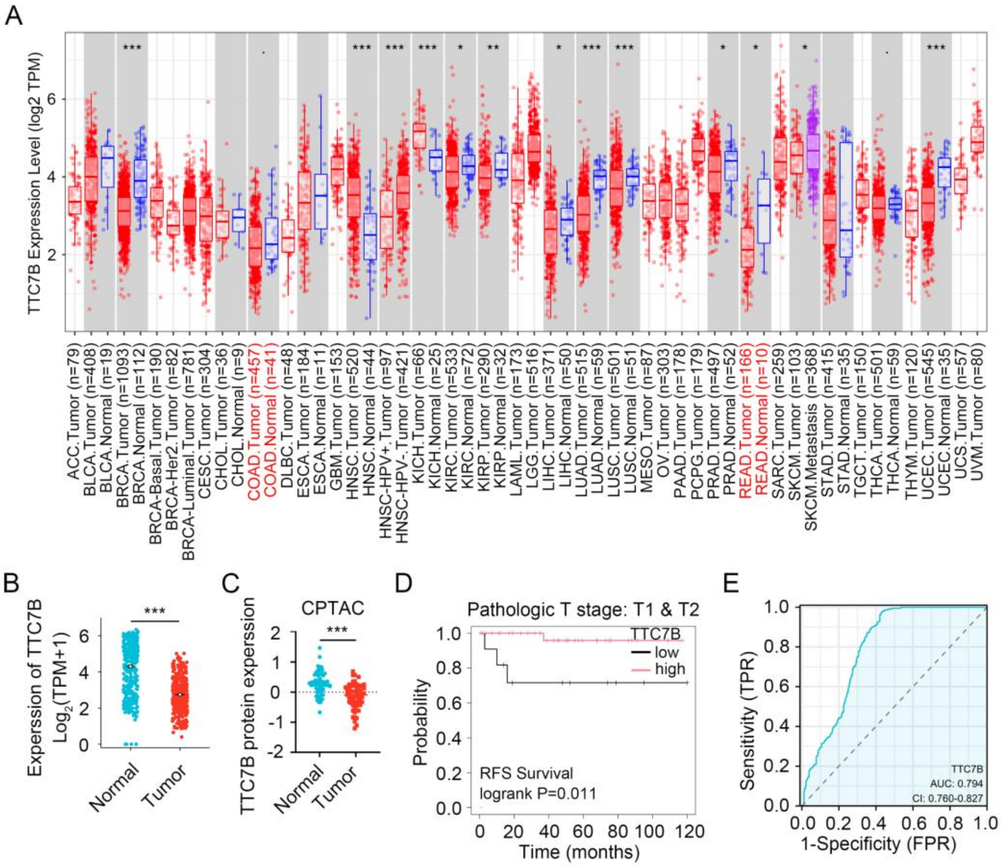

Figure S5. TTC7B expression is significantly reduced in colorectal cancer,

correlating with shorter RFS in CRC patients. (A) Analysis of TTC7B mRNA expression levels in various cancers and corresponding normal tissues using TCGA data from the TIMER website. (B) TTC7B mRNA expression levels in colorectal cancer tissues and normal tissues base on TCGA data. (C) TTC7B protein expression levels in colon cancer and adjacent normal tissues on CPTAC website. (D) Kaplan-Meier survival analysis examining the relationship between TTC7B expression and Recurrence-Free Survival (RFS) in CRC patients with pathological stages T1 and T2 in the TCGA database. (E) ROC curve evaluating the diagnostic value of TTC7B for CRC. Data are presented as mean ± SD. *P < 0.05, **P < 0.01, ***P < 0.001.

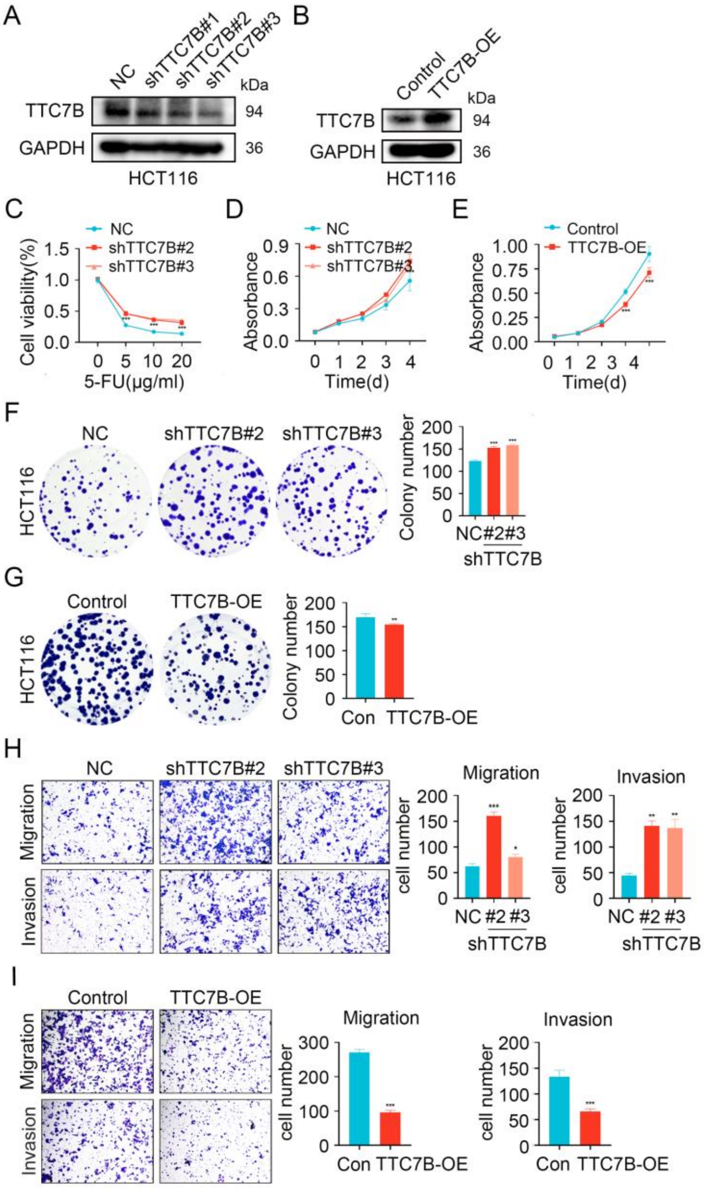

Figure S6. TTC7B inhibits chemotherapy resistance, proliferation, migration, and invasion. (A) TTC7B knockdown in HCT116 cells using three independent shRNAs. (B) Overexpression of TTC7B in HCT116 cells. (C) Cell viability evaluated following exposure to varying concentrations of 5-FU. (D-G) CCK8 and colony formation assays showing proliferation ability. (H-I) Transwell assay evaluating the migratory and

invasive capabilities. Data are presented as mean ± SD. *P < 0.05, **P < 0.01, ***P < 0.001.

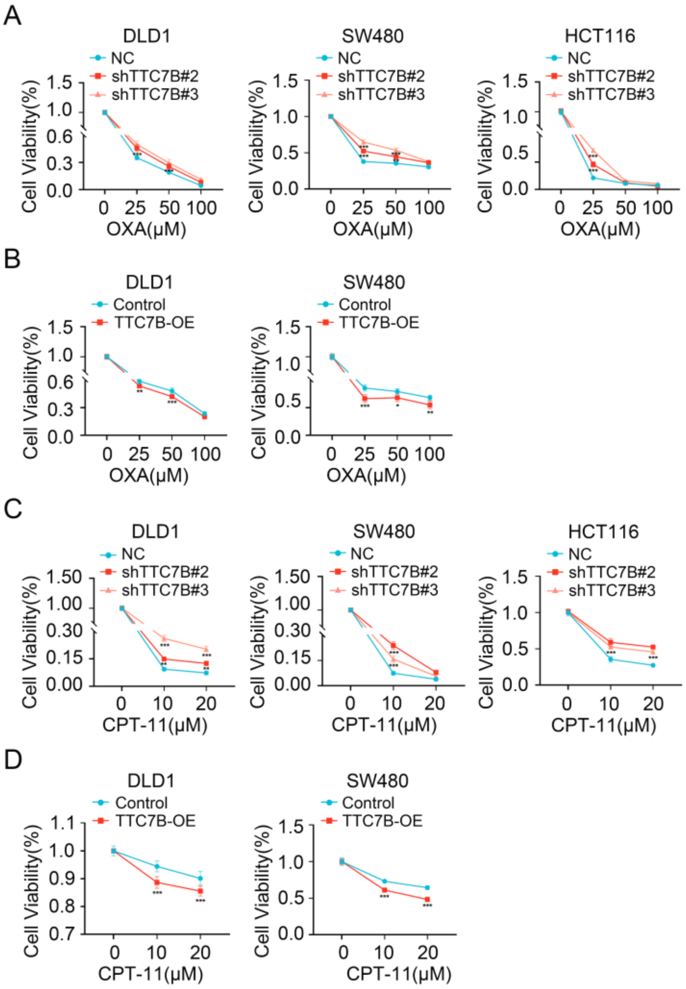

Figure S7. TTC7B inhibits chemoresistance in colorectal cancer cells. (A-D) Cell viability measured following exposure to varying concentrations of OXA (A-B) or CPT-11 (C-D). Data are presented as mean ± SD. *P < 0.05, **P < 0.01, ***P < 0.001.

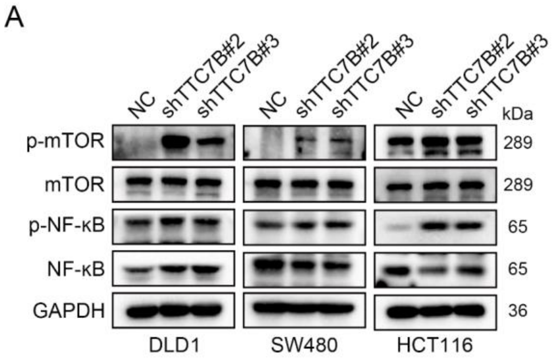

Figure S8. TTC7B inhibits the mTOR/NF-κB signaling pathway. (A) Detection of mTOR, NF-κB, and their phosphorylation levels in DLD1, SW480, and HCT116 cells with knockdown of TTC7B.

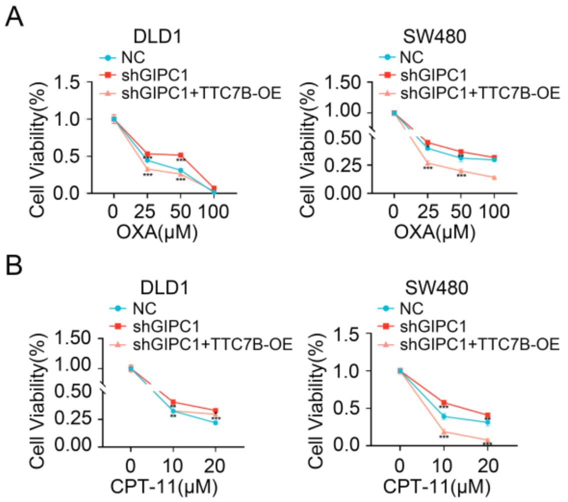

Figure S9. GIPC1 reverses chemoresistance in colorectal cancer through TTC7B. (A-B) Cell viability measured following exposure to varying concentrations of OXA (A) or CPT-11 (B). Data are presented as mean ± SD. *P < 0.05, **P < 0.01, and ***P < 0.001.

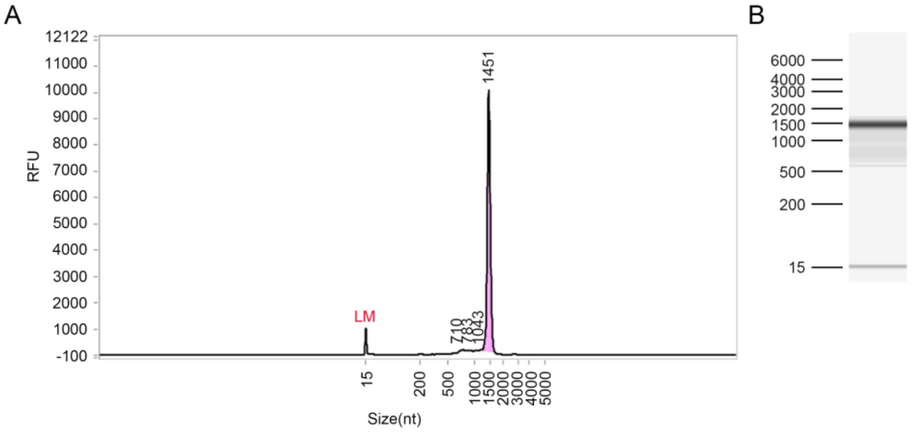

Figure S10. Analysis of GIPC1 mRNA. (A-B) Capillary electrophoresis analysis of GIPC1 mRNA.

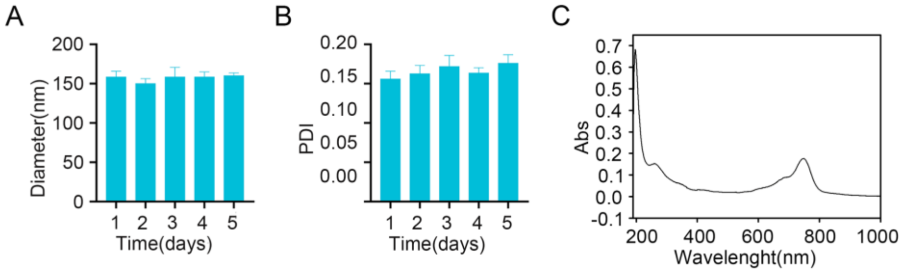

Figure S11. Characteristics of GIPC1-LNPs. (A-C) Average size (A), polydispersity index (PDI) (B), and UV absorbance value (C) of GIPC1-LNPs.

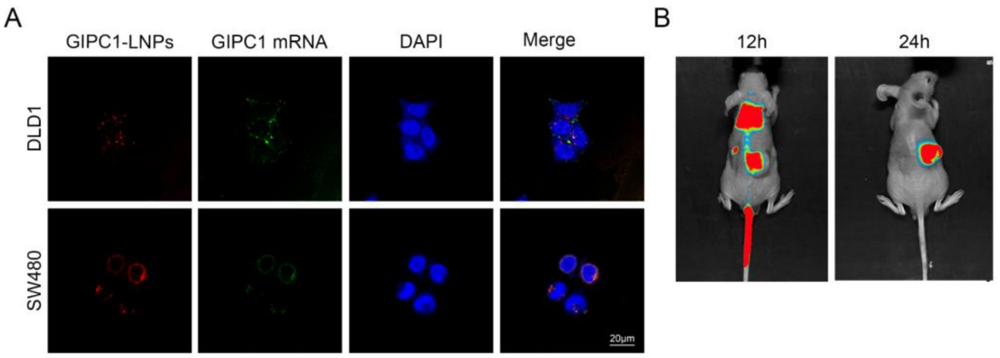

Figure S12. GIPC1-LNPs deliver mRNA to CRC cells and tumor tissues. (A) Representative fluorescence images of DIR-labeled LNPs (red) and cy3-labeled mRNA (green) in CRC cells, with nuclei co-stained with DAPI (blue). Scale bar = 20 μm. (B) Images at 12 hours and 24 hours after tail vein injection of DIR-labeled GIPC1-LNPs in CDX model mice.

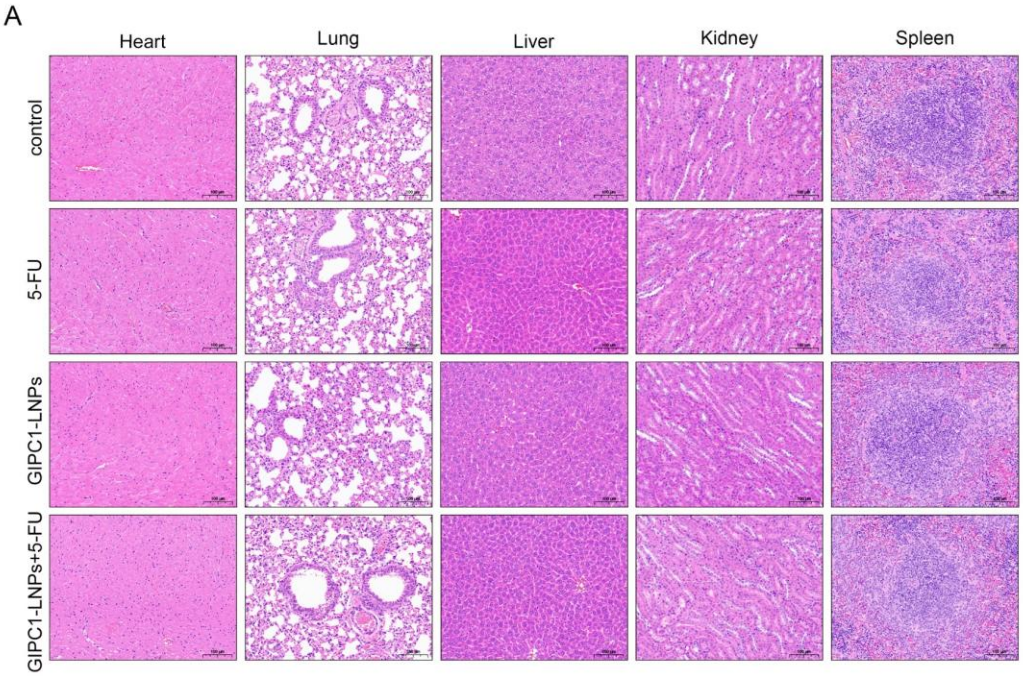

Figure S13. Systemic toxicity assessment of LNPs. (A) Histopathological examination of heart, lung, liver, spleen, and kidney sections was conducted in CDX model mice, utilizing H&E staining. Scale bar = 100 μm.
